# Poly(ester imide)s with Low Linear Coefficients of Thermal Expansion and Low Water Uptake (VII): A Strategy to Achieve Ultra-Low Dissipation Factors at 10 GHz

**DOI:** 10.3390/polym16050653

**Published:** 2024-02-28

**Authors:** Masatoshi Hasegawa, Taro Fukuda, Junichi Ishii

**Affiliations:** Department of Chemistry, Faculty of Science, Toho University, 2-2-1 Miyama, Funabashi 274-8510, Chiba, Japan

**Keywords:** poly(ester imide)s, polyimides, 2,6-naphthalene unit, heat resistance, linear coefficients of thermal expansion (CTE), water uptake, dielectric constants (DK), dissipation factors (DF), haze, 10 GHz, 5G, flexible printed circuits (FPC)

## Abstract

In this study, a series of ester-linked tetracarboxylic dianhydrides (TCDAs) with 2,6-naphthalene-containing longitudinally extended structures consisting of different numbers of aromatic rings (*N*_Ar_ = 6–8) was synthesized to obtain novel modified polyimides, poly(ester imide)s (PEsIs). These TCDAs were fully compatible with the conventional manufacturing processes of conventional polyimide (PI) systems. As an example, the PEsI film obtained from the ester-linked TCDA (*N*_Ar_ = 8) and an ester-linked diamine achieved unprecedented outstanding dielectric properties without the support of fluorinated monomers, specifically an ultra-low dissipation factor (tan *δ*) of 0.00128 at a frequency of 10 GHz (50% RH and 23 °C), in addition to an extremely high glass transition temperature (*T*_g_) of 365 °C, extremely low linear coefficient of thermal expansion (CTE) of 6.8 ppm K^−1^, suppressed water uptake (0.24%), requisite film ductility, and a low haze. Consequently, certain PEsI films developed in this study are promising candidates for heat-resistant dielectric substrates for use in 5G-compatible high-speed flexible printed circuit boards (FPCs). The chemical and physical factors denominating tan *δ* are also discussed.

## 1. Introduction

**Figure 1:**
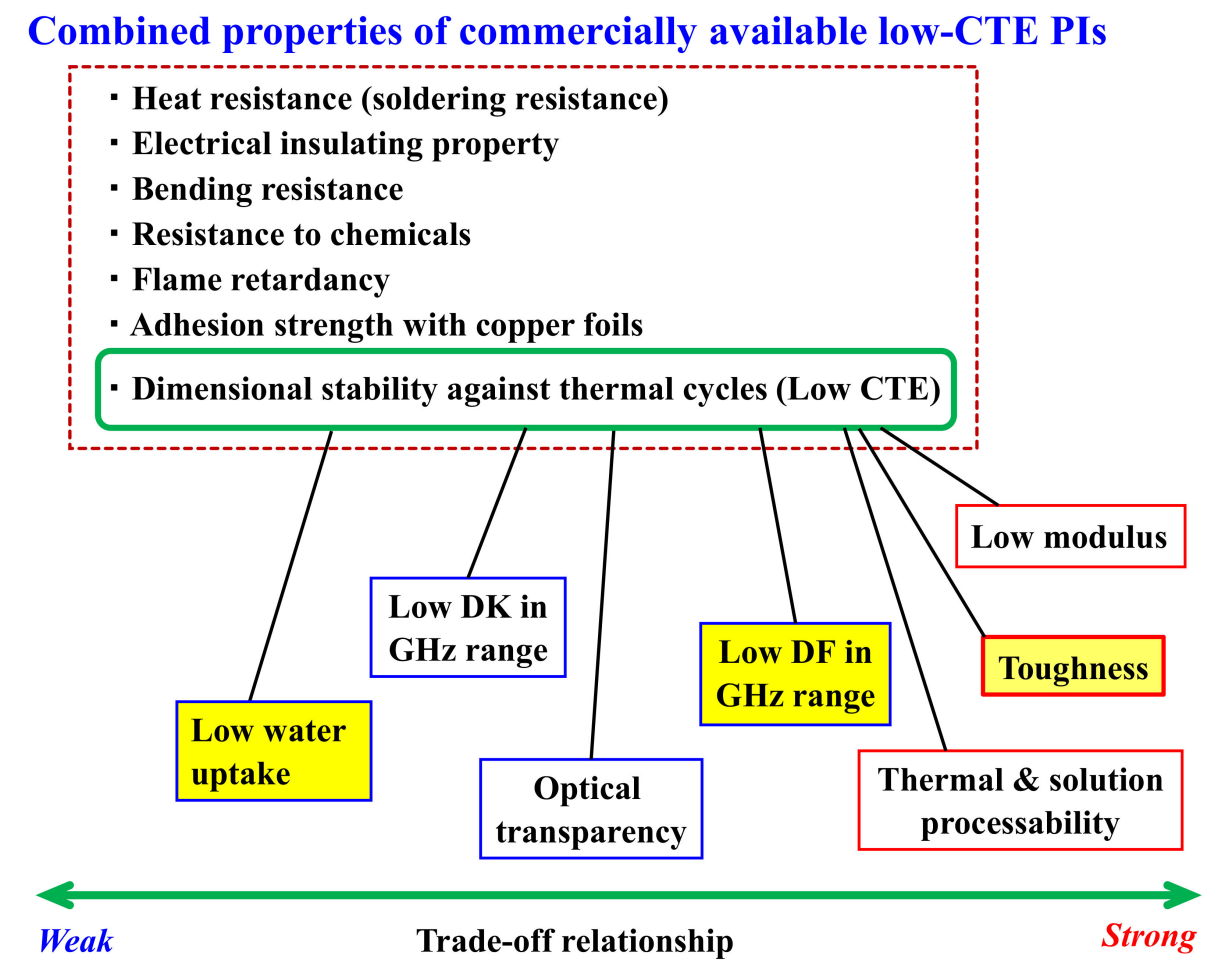
The combined properties of commercially available low-CTE polyimide films (surrounded by red broken line) and the difficulty in simultaneously achieving low CTE and other desired properties, relating to the trade-off in principle between them. The yellow-highlighted items represent the target properties of the materials developed in this study.

Wholly aromatic polyimides (PIs) are typical high-temperature polymers that have the highest physical [glass transition temperatures (*T*_g_)] and chemical heat resistance [thermal decomposition temperatures (*T*_d_)] among polymeric materials [1,2,3,4]. These films exhibit top-tier flame retardancy, high resistance to various chemicals, and excellent mechanical properties, in addition to outstanding electrical insulation reliability, which arises from their high resin purity characterized by the absence of ionic, metallic, and halogenic impurities; residual solvents; monomers; and unimidized portions [5,6,7,8,9]. Owing to their unique and complex properties (Figure 1), PIs have found widespread use as electrical insulation layers in a variety of electronic devices [10]. The rapid development of electronic devices has led to comprehensive studies in chemistry, physics, property improvement, functionalization, and applications [11,12,13,14,15]. Various commercial forms have emerged, including substrate-free rolled films, copper-clad laminates, pencil-compatible paper-like sheets, insulator-coated magnet wires, fibers, tubes, powder (or nanoparticles), spongy foams, molding/sintered compounds, photosensitive coatings, screen-printing inks, and prepregs. The highest demand among them is for rolled PI films, which are mainly used as high-temperature dielectric films in foldable circuits [typically referred to as flexible printed circuits (FPCs)].

The demand for FPCs has recently extended to include not only conventional electronic devices but also automotive electronic components, mobile phones and tablets, high-frequency antennas, transceiver modules, and medical and robot equipment. The most commonly used PI film for dielectric layers in conventional FPCs is the PMDA/4,4′-ODA-type KAPTON^®^ H film (USA, DuPont) [16] or similar types, derived from pyromellitic dianhydride (PMDA) and 4,4′-oxydianiline (4,4′-ODA). This PI film nearly satisfies the essential characteristics for conventional FPCs due to its combined properties.

FPCs are typically produced using the subtractive method, involving (1) the fabrication of single-sided or double-sided flexible copper clad laminates (FCCLs) through the lamination of a copper foil and a non-thermoplastic PI film such as KAPTON^®^ H film using strong adhesives (e.g., epoxy resin/acrylic rubber compounds) and (2) subsequent circuit formation via the fine pattern etching of the copper layers in the FCCLs using a photolithographic technique. Recently, the permissible restrictions for misalignment during FPC fabrication have become more severe. Hence, enhancing the dimensional stability of FPCs against heating–cooling cycles during their fabrication is crucial. One solution involves using pseudo-two-layered FCCLs by replacing conventional adhesives with dimensionally more stable and higher-*T*_g_ thermoplastic PIs (TPIs) or TPI-free two-layered FCCLs. These are prepared via the direct solution casting of the precursors of non-thermoplastic PIs onto the copper foil and their subsequent thermal imidization. The demand for FPCs obtained from these dimensionally stable FCCLs has notably increased. In addition, enhancing the thermal dimensional stability of the dielectric films in FPCs is essential. A direct strategy involves ensuring much higher *T*_g_s in dielectric films than the solder-reflow temperature (260 °C) and reducing their *XY*-direction linear coefficients of thermal expansion (CTEs) in the glassy temperature range (*T* < *T*_g_). Thereby, significant expansion–contraction during the thermal cycles can be suppressed. Improvements are sought for the CTE of KAPTON^®^ H film, higher than that of copper foils [16]. Thus, the demand for low-CTE PI films (e.g., UPILEX^®^-S [17] and KAPTON^®^ EN [18] as an improved version of KAPTON^®^ H) has increased. Achieving a low CTE and other required properties simultaneously poses challenges and often involves a trade-off relationship, as described later in Figure 1.

Additionally, dimensional stability against moisture/water absorption has also become important. KAPTON^®^ H film exhibits low dimensional stability against moisture/water absorption due to high water uptake (*W*_A_) and a high linear coefficient of humidity (hygroscopic) expansion (CHE) [16], linked to its high imide group content.

Our previous studies on modified PIs, specifically poly(ester imide)s (PEsIs), obtained from specific *para*-linked monomers with longitudinally extended structures via ester connecting groups, demonstrated effectiveness in reducing *W*_A_ responsible for hygroscopic expansion, while maintaining very high *T*_g_s, extremely low CTEs, and the advantages for their FPC applications; the effects of substituents [19,20], the structural extension of tetracarboxylic dianhydrides [21] and diamines [22], and asymmetric structures [23] on CTE, *T*_g_, *W*_A_, and mechanical and dielectric properties have been investigated. In contrast, earlier PEsIs, which were synthesized via the direct polycondensation of imide-containing bisphenols with common dicarbonyl chlorides [24] or imide-containing dicarbonyl chlorides with common bisphenols [25,26], had no potential applications as dielectric substrates in FPCs, probably owing to the difficulty in obtaining high-quality films with a low CTE. Therefore, no attention has been paid to *W*_A_ and the dielectric properties of the earlier PEsIs.

To support the current 5G shift of the network communication standard enabling high-speed, high-capacity, low-delay, and simultaneousmultiple-connection communications, it has become indispensable to significantly reduce the dissipation factor (DF, tan *δ*) of the dielectric films in specific FPCs (e.g., FPC for connecting the antenna-in-package to the main board in mobile phones). However, a serious problem arises as conventional PI films have high tan *δ* values originating from the high content of highly polarized imide groups (behaving as permanent dipoles) present in their structures. This is responsible for orientational polarization induced by high-frequency electric fields in the GHz range (e.g., tan *δ* = 0.0085 at 1 GHz for KAPTON^®^ H film [16]). Therefore, dielectric film materials possessing high *T*_g_, low CTE, and low tan *δ* simultaneously have become strongly required for the above-mentioned specific FPCs. However, producing such materials has been very difficult so far.

Adsorbed and absorbed water in dielectric films significantly deteriorate their dielectric properties owing to the high *ε*_r_ and high tan *δ* of water [27]. Thus, the low-*W*_A_ PEsI systems that we have studied so far are expected to be advantageous for controlling their DK and DF. Certain PEsIs were effective in reducing the DF and *W*_A_ while maintaining high *T*_g_ and low CTE properties [20,21,22,23].

In this study, we propose a detailed molecular design for dramatically reducing the DF without sacrificing the afore-mentioned other required properties and report the performance of the novel PEsI films developed. The study also discusses the factors influencing DF.

## 2. Experimental Section

### 2.1. Materials

#### 2.1.1. Monomer Synthesis

(a) TA-26NAHNA (*N*_Ar_ = 8)

**Figure 2:**
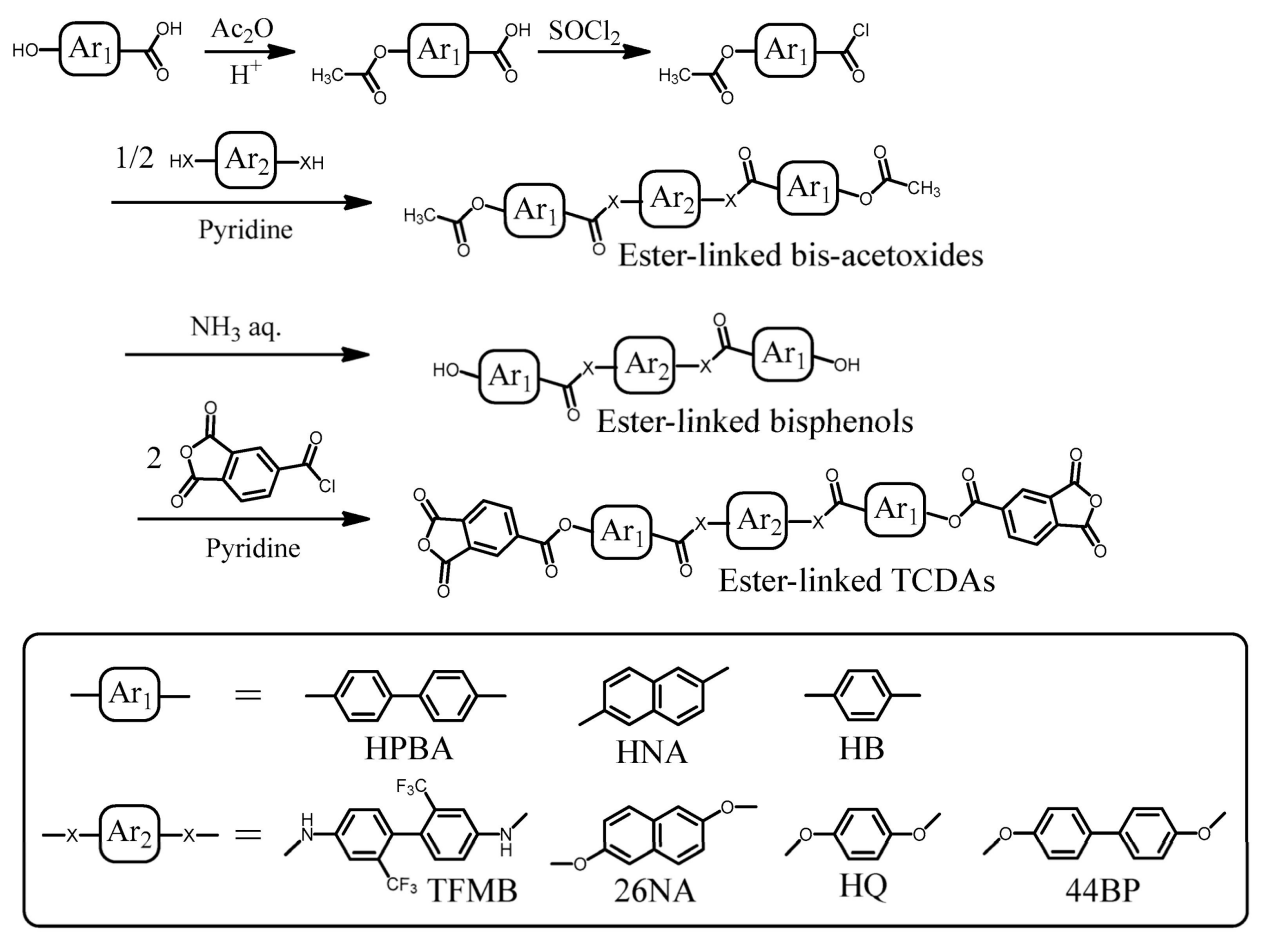
Reaction scheme for the synthesis of ester-linked tetracarboxylic dianhydrides with longitudinally extended structures.

A novel ester-linked tetracarboxylic dianhydride (TCDA) with *N*_Ar_ = 8 (TA-26NAHNA) was synthesized according to the reaction scheme shown in Figure 2. The detailed procedures at each step are shown below.

6-Acetoxy-2-naphthalenecarboxylic acid (AcNA). In a 300 mL three-necked flask, the mixture of 6-hydroxy-2-naphthalenecarboxylic acid (HNA, 17.11 g, 912 mmol), acetic anhydride (70 mL), and three drops of conc. sulfuric acid as a catalyst was refluxed at 100 °C for 3 h in a nitrogen atmosphere, and the resulting clear solution was cooled to room temperature. The cream-colored precipitate formed was collected by filtration, washed with water and toluene, and dried at 120 °C for 12 h under vacuum (yield: 78%). 

**Scheme 1:**
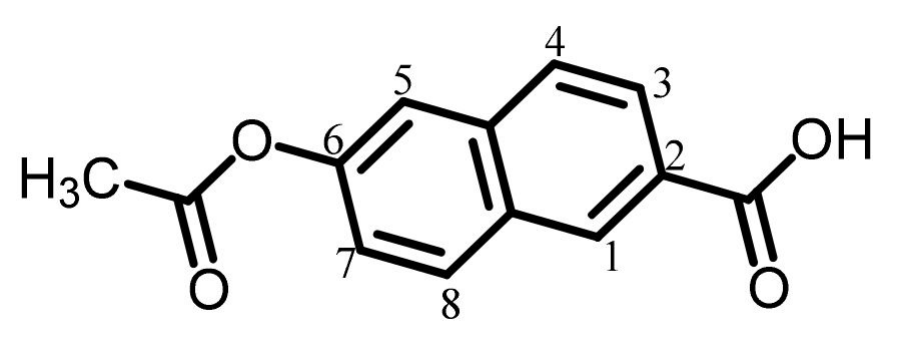
Structure of AcNA with numbering.

The analytical data of this product are as follows. Melting point: 203 °C (TG-DTA). FT-IR (KBr plate method, cm^−1^): 3053 (C_Ar_–H stretching vibration band), 2987/2845 (C_Aliph_–H), 2684 (hydrogen-bonded COOH group, O–H), 1761 (acetoxy group, C=O), 1680 (hydrogen-bonded COOH group, C=O), 1223 (acetoxy group, C–O). ^1^H-NMR (400 MHz, dimethyl sulfoxide (DMSO)-*d*_6_, *δ*, ppm): 13.11 [s, 1H (relative integrated intensity: 0.90H), COOH], 8.64 [s, 1H (1.15H), 1-proton of the central 2,6-naphthalene (26NA) unit], 8.18 [d, 1H (1.07H), *J* = 8.9 Hz, 8-proton of 26NA], 8.03–7.99 [m, 2H (2.27H), 3- and 4-protons of 26NA], 7.77 [d, 1H (1.15H), *J* = 2.2 Hz, 5-proton of 26NA], 7.42 (dd, 1H (1.09H), *J* = 8.9, 2.2 Hz, 7-proton of 26NA), 2.34 [s, 3H (3.00H), CH_3_]. The results confirm that the product is the desired acetylated compound (AcNA) shown in Scheme 1.

6-Acetoxy-2-naphthalenecarbonyl chloride (AcNAC). In a 300 mL three-necked flask, the mixture of AcNA (15.37 g, 49 mmol), a large excess of thionyl chloride (30 mL), and five drops of *N*,*N*-dimethylformamide (DMF) as a catalyst was refluxed at 80 °C for 3 h in a nitrogen atmosphere. To the clear solution obtained, benzene was added, and an excess of thionyl chloride was azeotropically distilled off under a reduced pressure. The yellow precipitate formed was recrystallized from cyclohexane and dried at 60 °C for 12 h under vacuum (yield: 78%). 

**Scheme 2:**
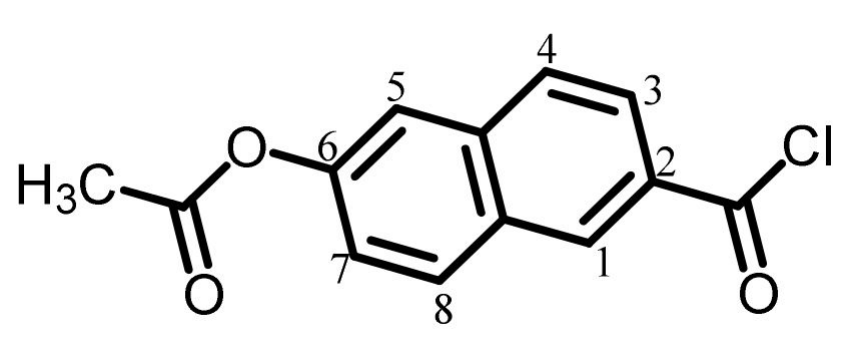
Structure of AcNAC with numbering.

The analytical data of this product are as follows. Melting point: 127 °C (TG-DTA). FT-IR (KBr plate method, cm^−1^): 3063 (C_Ar_–H), 1741 (OCOCH_3_ + COCl groups, C=O). The disappearance of the infrared bands at ~2600 and 1680 cm^−1^ originating from the COOH groups was confirmed. ^1^H-NMR (400 MHz, CDCl_3_, *δ*, ppm): 8.74 [s, 1H (1.04H), 1-proton of 26NA], 8.08–8.02 [m, 2H (2.18H), 8- and 3-protons of 26NA], 7.88 [d, 1H (1.09H), *J* = 8.7 Hz, 4-proton of 26NA], 7.65 [d, 1H (1.04H), *J* = 2.0 Hz, 5-proton of 26NA], 7.37 [dd, 1H (1.06H), *J* = 8.9, 2.2 Hz, 7-proton of 26NA], 2.38 [s, 3H (3.00H), CH_3_]. The disappearance of the COOH proton signal at *δ* = 13.11 ppm was confirmed. The results confirm that the product is the desired chlorinated compound (AcNAC) shown in Scheme 2.

AcNA26NA (diacetoxy compound). In a 100 mL flask, 2,6-dihydroxynaphthalene (26DHNA, 4.80 g, 29.9 mmol) was dissolved in dehydrated *γ*-butyrolactone (GBL, 20 mL) in the presence of pyridine (8 mL, ~100 mmol) as an acid acceptor, and the solution was sealed using a septum rubber stopper (solution-A). In another 500 mL flask, AcNAC (16.93 g, 68 mmol) was dissolved in dehydrated GBL (210 mL), and the solution was sealed with a septum rubber stopper (solution-B). Solution-A was gradually added to solution-B at room temperature with a syringe, and the reaction mixture was stirred at room temperature for 12 h. The precipitate yielded was collected by filtration, washed with toluene and GBL to remove an excess of AcNAC, and subsequently, washed with a large quantity of water to remove the by-product (pyridine-HCl salt). The cream-colored product was dried at 160 °C for 12 h under vacuum (yield: 82%). Thin-layer chromatography (TLC) suggested that this product contains no undesirable mono-esterified compound. 

**Scheme 3:**
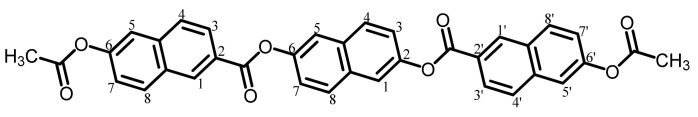
Structure of AcNA26NA with numbering.

The analytical data of this product are as follows. Melting point: 220 °C (TG-DTA). FT-IR (KBr plate method, cm^−1^): 3068 (C_Ar_–H), 1757 (OCOCH_3_ groups, C=O), 1736 (aromatic ester group, C=O). ^1^H-NMR (400 MHz, DMSO-*d*_6_, *δ*, ppm): 8.97 [d, 2H (2.00H), *J* = 0.84 Hz, 1,1′-protons of the terminal 2,6-NA units], 8.32 [d, 2H (1.97H), *J* = 9.2 Hz, 8,8′-protons of the terminal 26NA], 8.22 [dd, 2H (1.91H), *J* = 8.6, 1.7 Hz, 3,3′-protons of the terminal 26NA], 8.15–8.10 [m, 4H (4.04H), 4,4′-protons of the terminal 26NA + 4,8-protons of the central 26NA], 8.02 [d, 2H (1.97H), *J* = 2.4 Hz, 5,5′-protons of the terminal 26NA], 7.86 [d, 2H (2.01H), *J* = 2.4 Hz, 1,5-protons of the central 26NA], 7.62 [dd, 2H (1.95H), *J* = 8.9, 2.4 Hz, 7,7′-protons of the terminal 26NA], 7.50 [dd, 2H (1.97H), *J* = 8.8, 2.3 Hz, 3,7-protons of the central 26NA], 2.37 [s, 6H (5.94H), CH_3_]. The results confirm that the product is the desired diacetoxy compound (AcNA26NA) shown in Scheme 3.

26NAHNA (Bisphenol). In a 500 mL flask, the afore-mentioned AcNA26NA (4.55 g, 8.4 mmol) was dissolved in dehydrated *N*-methyl-2-pyrrolidone (NMP, 50 mL) by soft-heating for a short time. To the solution cooled at 0 °C, a conc. NH_3_ aqueous solution (28 wt%, 40 mL) was gradually added, and the reaction mixture was stirred for 20 min to complete hydrolysis of the terminal acetoxy groups. The cream-colored precipitate formed by subsequent neutralization with a 1N-HCl aqueous solution (60 mL) was collected by filtration, washed with water, and dried at 160 °C for 12 h under vacuum (yield: 94%). 

**Scheme 4:**
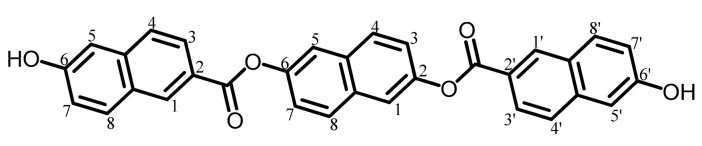
Structure of 26NAHNA with numbering.

The analytical data of this product are as follows. Melting point: 328 °C (TG-DTA). FT-IR (KBr plate method, cm^−1^): 3419 (O–H), 3069 (C_Ar_–H), 1717 (ester groups, C=O). In addition, the disappearance of the C=O stretching vibration band at 1757 cm^−1^ originating from the acetoxy groups was confirmed. ^1^H-NMR (400 MHz, DMSO-*d*_6_, *δ*, ppm): 10.33 [s, 2H (2.00H), OH], 8.79 [d, 2H (1.95H), *J* = 1.4 Hz, 1,1′-protons of the terminal 2,6-NA units], 8.10–8.05 [m, 6H (5.94H), 8,8′- + 3,3′- + 4,4′-protons of the terminal 26NA], 7.97 [d, 2H (1.87H), *J* = 2.4 Hz, 5,5′-protons of the terminal 26NA], 7.88 [d, 2H (1.99H), *J* = 8.8 Hz, 4,8-protons of the central 26NA], 7.58 [dd, 2H (1.98H), *J* = 8.5, 2.5 Hz, 7,7′-protons of the terminal 26NA], 7.27–7.22 [m, 4H (4.06H), 1,5- + 3,7-protons of the central 26NA]. In addition, the disappearance of the proton signal at 2.37 ppm for the CH_3_ groups was confirmed. The results indicate that this product is the desired bisphenol (26NAHNA) shown in Scheme 4.

TA-26NAHNA (TCDA). In a 100 mL flask, the above-mentioned ester-linked bisphenol (26NAHNA, 3.58 g, 7.16 mmol) was dissolved in dehydrated NMP (20 mL) in the presence of pyridine (5 mL, 63 mmol), and the solution was sealed with a septum rubber stopper (solution-C). In another 200 mL flask, an excess amount of trimellitic anhydride chloride (TMAC, 6.34 g, 30 mmol) was dissolved in dehydrated NMP (20 mL), and the solution was sealed with a septum rubber stopper (solution-D). Solution-C was gradually added to solution-D at room temperature using a syringe, and the reaction mixture was stirred at room temperature for 12 h. The precipitate yielded was collected by filtration, washed with toluene and GBL to remove an excess of TMAC, and subsequently, washed with a large quantity of water to remove the by-product (pyridine-HCl salt). The pale-brown product was dried at 200 °C for 12 h under vacuum (yield: 68%). 

**Scheme 5:**
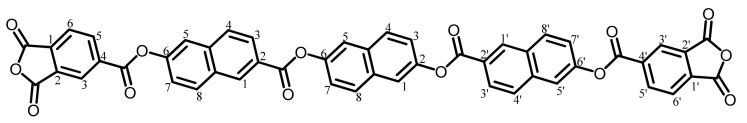
Structure of TA-26NAHNA with numbering.

The analytical data of this product are as follows. Melting point: 288 °C (DSC). FT-IR (KBr plate method, cm^−1^): 3065 (C_Ar_–H), 1858/1784 (acid anhydride groups, C=O), 1737 (ester groups, C=O). In addition, the O–H stretching vibration band at 3419 cm^−1^ completely disappeared. ^1^H-NMR (400 MHz, DMSO-*d*_6_, *δ*, ppm): 9.04 [d, 2H (2.00H), *J* = 1.8 Hz, 1,1′-protons of the O–NA–CO units], 8.73–8.70 [m, 4H (4.17H), 3,3′- + 6,6′-protons of the phthalic anhydride (PAn) units], 8.42 [d, 2H (1.83H), *J* = 8.6 Hz, 8,8′-protons of the 6-O–NA–2-CO units], 8.33 [dd, 2H (1.65H), *J* = 7.7, 0.8 Hz, 5,5′-protons of PAn], 8.27 [dd, 2H (2.37H), *J* = 8.4, 1.7 Hz, 3,3′-protons of the O–NA–CO units], 8.21 [d, 2H (2.18H), *J* = 8.6 Hz, 4,4′-protons of the O–NA–CO units], 8.14–8.12 [m, 4H (3.88H), 5,5′-protons of the O–NA–CO units + 1,5-protons of the central 26NA unit], 8.04 [d, 2H (2.14H), *J* = 8.6 Hz, 4,8-protons of the central 26NA], 7.77 [dd, 2H (1.74H), *J* = 8.7, 2.1 Hz, 7,7′-protons of the O–NA–CO units], 7.65 [dd, 2H (2.38H), *J* = 8.6, 2.1 Hz, 3,7-protons of the central 26NA]. In addition, the disappearance of the OH proton signal at 10.33 ppm was confirmed. Elemental analysis, Anal. Calcd. (%) for C_50_H_24_O_14_ (848.73): C, 70.76; H, 2.85. Found: C, 70.61; H, 3.06. The results correspond to the structure of the desired ester-linked TCDA (TA-26NAHNA, *N*_Ar_ = 8) shown in Scheme 5.

(b) TA-26NAHB (*N*_Ar_ = 6)

Another ester-linked TCDA (TA-26NAHB) was synthesized according to the reaction scheme shown in Figure 2 using 4-hydroxybenzoic acid (HBA) instead of HNA in the same procedures as mentioned above. 

**Scheme 6:**
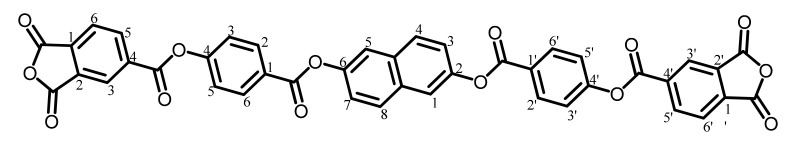
Structure of TA-26NAHB with numbering.

The analytical data of the product are as follows. Melting point: 283 °C (DSC). FT-IR (KBr plate method, cm^−1^): 3111/3068 (C_Ar_–H), 1860/1781 (acid anhydride groups, C=O), 1747/1736 (ester groups, C=O), 1509 (1,4-phenylene groups). ^1^H-NMR (400 MHz, DMSO-*d*_6_, *δ*, ppm): 8.70–8.68 [m, 4H (4.04H), 3,3′- + 6,6′-protons of PAn], 8.35 [d, 4H (3.92H), *J* = 8.9 Hz, 2,2′,6,6′-protons of the 4-O-Ph-1-CO units], 8.31 [dd, 2H (2.11H), *J* = 7.6, 1.0 Hz, 5,5′-protons of PAn], 8.10 [d, 2H (2.13H), *J* = 8.8 Hz, 4,8-protons of the central 26NA], 7.98 [d, 2H (2.00H), *J* = 2.4 Hz, 1,5-protons of the central 26NA], 7.70 [d, 4H (3.86H), *J* = 8.8 Hz, 3,3′,5,5′-protons of the O-Ph-CO units], 7.60 [dd, 2H (1.93H), *J* = 8.8, 2.6 Hz, 3,7-protons of the central 26NA]. Elemental analysis, Anal. Calcd. (%) for C_42_H_20_O_14_ (748.61): C, 67.39; H, 2.69. Found: C, 67.71; H, 2.89. The results correspond to the structure of the desired ester-linked TCDA (*N*_Ar_ = 6) shown in Scheme 6.

(c) TA-HQHNA (*N*_Ar_ = 7)

TA-HQHNA was synthesized according to the reaction scheme shown in Figure 2 using hydroquinone (HQ) instead of 26DHNA in the same procedures as mentioned above. 

**Scheme 7:**
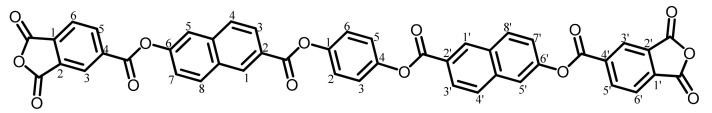
Structure of TA-HQHNA with numbering.

The analytical data of the product are as follows. Melting point: 306 °C (DSC). FT-IR (KBr plate method, cm^−1^): 3072 (C_Ar_–H), 1853/1779 (acid anhydride groups, C=O), 1742 (ester groups, C=O), 1506 (1,4-phenylene group). ^1^H-NMR (400 MHz, DMSO-*d*_6_, *δ*, ppm): 8.99 [s, 2H (2.13H), 1,1′-protons of the 6-O–NA–2-CO units], 8.73–8.69 [m, 4H (3.86H), 3,3′- + 6,6′-protons of PAn], 8.41 [d, 2H (2.07H), *J* = 9.0 Hz, 8,8′-protons of the O–NA–CO units], 8.33 [dd, 2H (2.22H), *J* = 7.8, 0.7 Hz, 5,5′-protons of PAn], 8.25–8.20 [m, 4H (4.19H), 3,3′- + 4,4′-protons of the O–NA–CO units], 8.14 [d, 2H (2.12H), *J* = 1.9 Hz, 5,5′-protons of the O–NA–CO units], 7.76 [dd, 2H (2.11H), *J* = 8.7, 2.3 Hz, 7,7′-protons of the O–NA–CO units], 7.52 [s, 4H (4.00H), 2,3,5,6-protons of the central HQ]. Elemental analysis, Anal. Calcd. (%) for C_46_H_22_O_14_ (798.67): C, 69.18; H, 2.78. Found: C, 68.78; H, 3.13. The results correspond to the structure of the desired ester-linked TCDA (TA-HQHNA, *N*_Ar_ = 7) shown in Scheme 7.

(d) TA-TFMBHPBA (*N*_Ar_ = 8)

TA-TFMBHPBA was synthesized from a 2,2′-bis(trifluoromethyl)benzidine (TFMB)-based bisphenol and TMAC according to the reaction scheme shown in Figure 2 in the same way as mentioned above. 

**Scheme 8:**
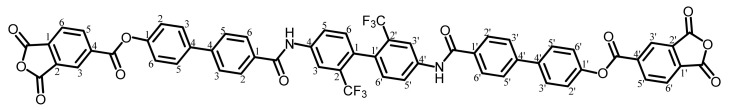
Structure of TA-TFMBHPBA with numbering.

The analytical data of the product are as follows. Melting point: 379 °C (DSC). FT-IR (KBr plate method, cm^−1^): 3394 (amide groups, N–H), 3106/3069 (C_Ar_–H), 1864/1846/1776 (acid anhydride groups, C=O), 1738 (ester groups, C=O), 1665/1528 (amide groups, C=O), 1493 (1,4-phenylene groups). ^1^H-NMR (400 MHz, DMSO-*d*_6_, *δ*, ppm): 10.73 [s, 2H (2.00H), NHCO], 8.69–8.65 [m, 4H (4.05H), 3,3′,6,6′-protons of PAn], 8.40 [d, 2H (1.97H), *J* = 0.84 Hz, 3,3′-protons of the central TFMB unit], 8.31 [d, 2H (1.95H), *J* = 7.4 Hz, 5,5′-protons of PAn], 8.15–8.13 [m, 6H (6.19H), 2,2′,6,6′-protons of the Ph–1-CO units + 5,5′-protons of the TFMB unit], 7.96–7.92 [m, 8H (8.11H), 3,3′,5,5′-protons of the Ph–CO units + 3,3′,5,5′-protons of the 1-O–Ph units], 7.56 [d, 4H (3.96H), *J* = 8.6 Hz, 2,2′,6,6′-protons of the O–Ph units], 7.42 [d, 2H (1.95H), *J* = 8.6 Hz, 6,6′-protons of the TFMB unit]. Elemental analysis, Anal. Calcd. (%) for C_58_H_30_O_12_N_2_F_6_ (1060.87): C, 65.67; H, 2.85; N, 2.64. Found: C, 65.32; H, 2.92; N, 2.69. The results correspond to the structure of the desired ester-linked TCDA (TA-TFMBHPBA, *N*_Ar_ = 8) shown in Scheme 8.

#### 2.1.2. Common Monomers and Raw Materials

**Figure 3:**
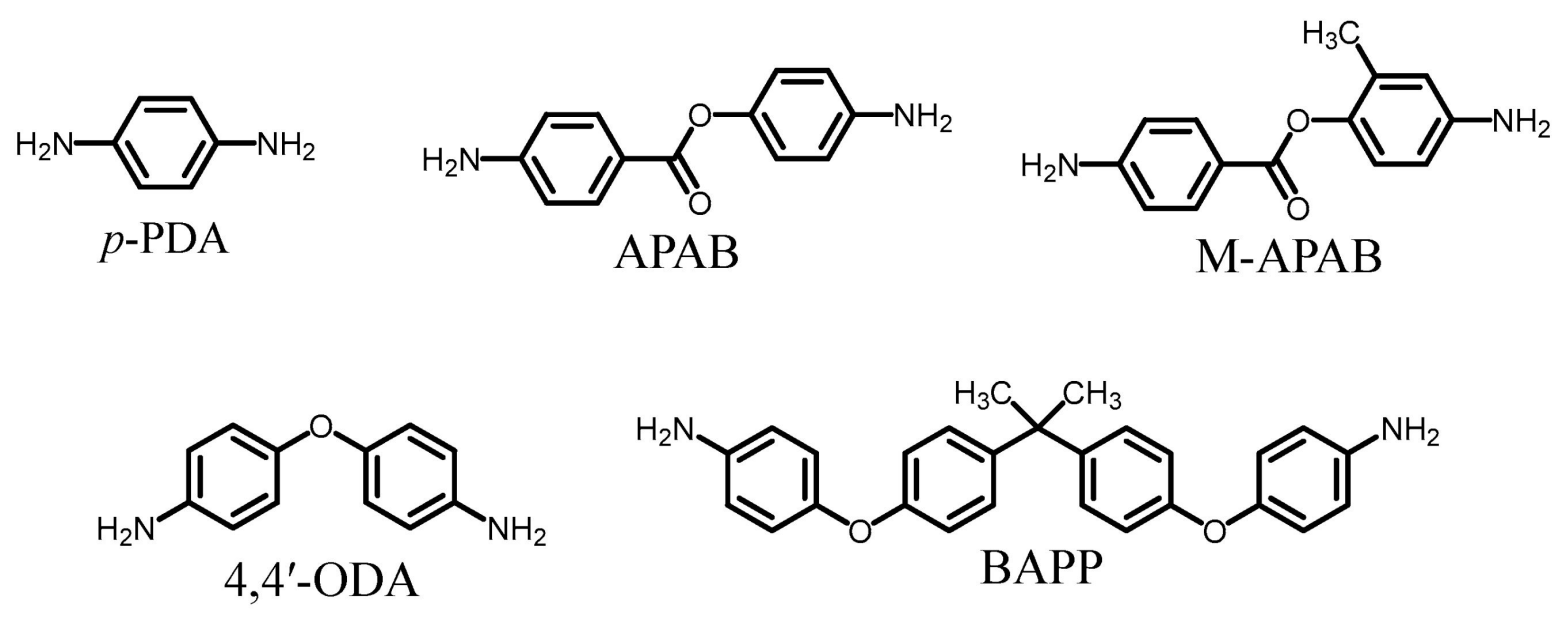
Structures and abbreviations of the diamines used in this study.

The molecular structures of the common monomers used in this study are shown in Figure 3. The sources and melting points of the common monomers and raw materials used in this study are listed in Table 1 and Table 2.

#### 2.1.3. Polyaddition and Thermal Imidization

**Figure 4:**
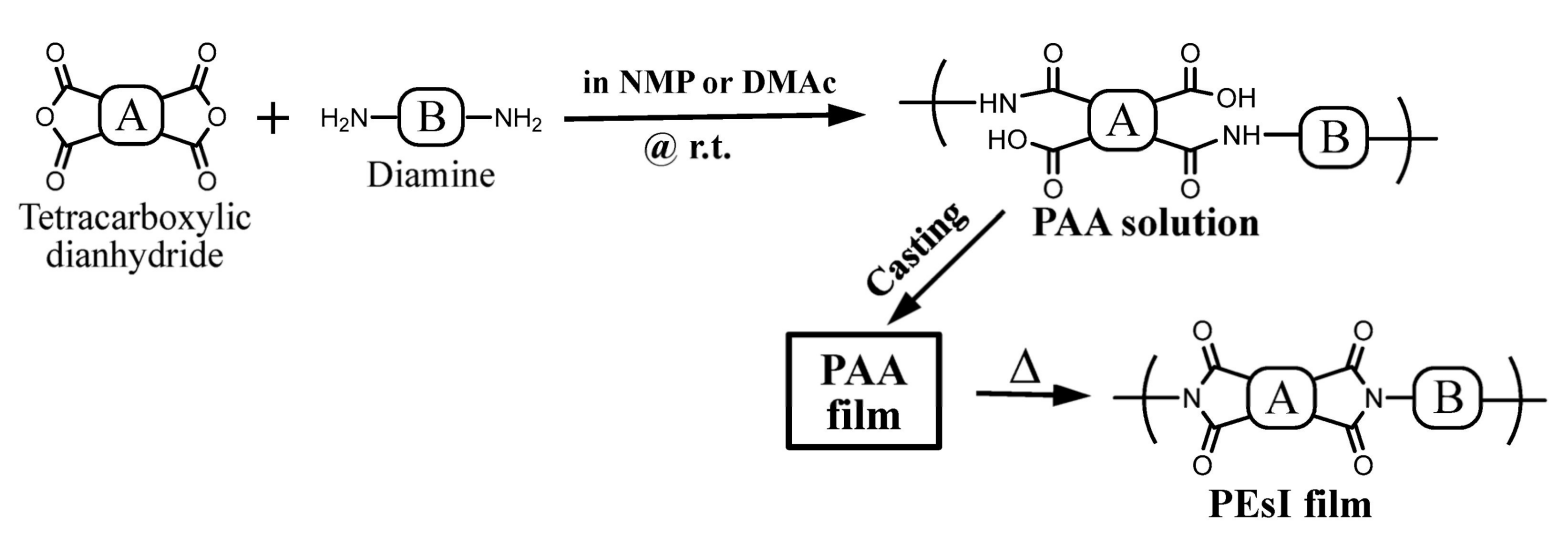
Schemes of polyaddition, solution casting of PAAs, and thermal imidization for PEsI film preparation.

The PEsI films were prepared via the equimolar polyaddition of tetracarboxylic dianhydrides and diamines, solution casting of the resulting PAAs, and thermal imidization of the PAA cast films, according to the scheme shown in Figure 4.

A typical procedure for the polyaddition is as follows. In a sealed vial, tetracarboxylic dianhydride powder (2 mmol) was added to an NMP solution of a diamine (2 mmol) in one portion with continuous magnetic stirring, and the reaction mixture (initial solid content: 25 or 30 wt%) was stirred at room temperature until it became homogeneous and reached the maximum solution viscosity (typically after 72 h). The reaction mixture was as appropriate gradually diluted with a minimal quantity of the same solvent to ensure effective magnetic stirring.

**Figure 5:**
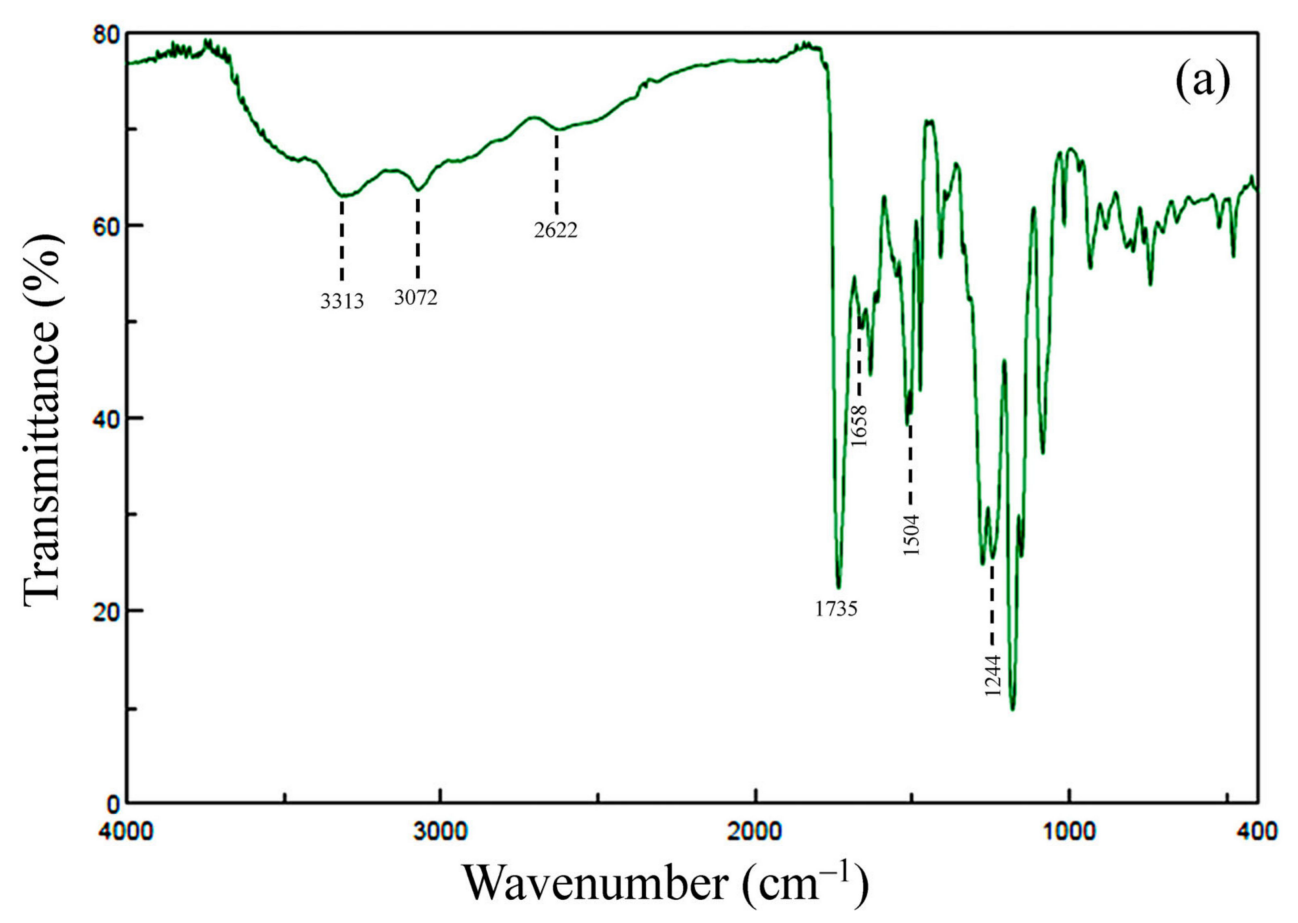

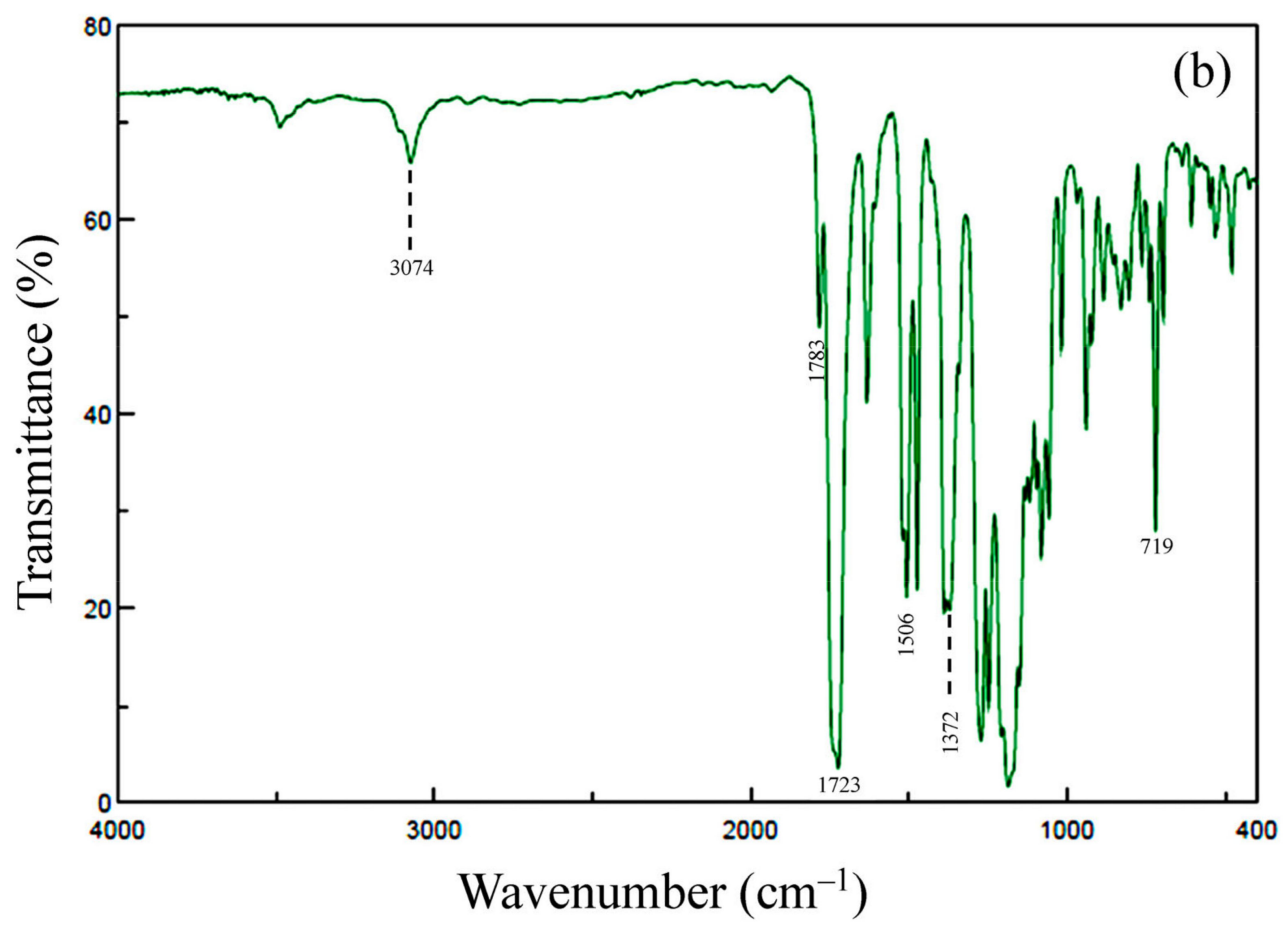
FT-IR spectra of thin films for TA-HQHNA/*p*-PDA: (**a**) PAA and (**b**) PEsI.

The formation of PAAs was confirmed by the transmission-mode FT-IR spectra of the separately prepared thin cast films (4–5 μm thick) with a non-uniform thickness to erase the interference fringes. A typical spectrum is shown in Figure 5a. This shows the following IR bands (cm^−1^): 3313 (amide groups, N–H stretching vibration band), 3072 (C_Ar_–H), 2622 (hydrogen-bonded COOH groups, O–H), 1735 (ester groups, C=O), 1658 (amide groups, C=O), 1504 (1,4-phenylene units), and 1244 (ester groups, CO–O). In addition, the disappearance of the IR band at 1860 cm^−1^ for the acid anhydride groups of the TCDA was confirmed. Thus, the spectrum corresponds to the structure of the desired PAA.

The PEsI films (typically, 25 μm thick) were prepared via thermal imidization of the PAA cast films as follows. The PAA solutions were bar-coated on a glass substrate and dried at 80 °C for 3 h in an air-convection oven. The cast films were thermally imidized at 250 °C for 1 h + 350 °C for 1 h on the substrate under vacuum and annealed at 350–360 °C for 1 h under vacuum without the substrate to remove residual stress. In some cases, these thermal conditions were adjusted to obtain better-quality flexible films without residual strain.

The completion of thermal imidization was confirmed by the FT-IR spectra of the separately prepared thin films. A typical spectrum is shown in Figure 5b. This shows the following IR bands (cm^−1^): 3074 (C_Ar_–H), 1783 (imide groups, C=O), 1723 (imide + ester groups, C=O), 1506 (1,4-phenylene units), 1372 (imide group, *N*–C_Ar_), and 719 (imide groups, ring deformation). In addition, the IR bands characteristic of the PAAs (e.g., the amide C=O stretching band at 1658 cm^−1^ and the hydrogen-bonded carboxylic acid O–H stretching band at 2622 cm^−1^) completely disappeared. Thus, the spectrum corresponds to the structure of the desired PEsI.

In this study, the PAA and the corresponding PEsI systems are abbreviated using the symbols of the TCDAs (A) and diamines (B) as A/B for homopolymers and A/B1;B2 for copolymers.

### 2.2. Measurements and Characterization

#### 2.2.1. Structural Characterization of Ester-Linked TCDAs

The chemical structures of the ester-linked TCDAs synthesized in this study and their intermediates were characterized by FT-IR (JASCO, Tokyo, Japan, FT/IR-4X infrared spectrometer), ^1^H-NMR spectroscopy (JEOL, Tokyo, Japan, ECP400), and elemental analysis (J-Science Lab, Kyoto, Japan, Micro Corder JM10). Their melting points were determined from the endothermic peak temperatures by differential scanning calorimetry (Rigaku, Tokyo, Japan, DSC8231) or differential temperature analysis (Netzsch Japan, Yokohama, Japan, TG-DTA2000S) with a heating rate of 5 °C min^−1^ in a nitrogen atmosphere.

#### 2.2.2. Inherent Viscosities

The reduced viscosities (*η*_red_) of the PEsI precursors [poly(amic acid)s, PAAs], which can be practically regarded as their *η*_inh_, were measured at a solid content of 0.5 wt% at 30 °C in NMP on an Ostwald viscometer.

#### 2.2.3. Linear Coefficients of Thermal Expansion (CTE)

The CTE values of the PEsI films (specimen size: 20 mm long, 5 mm wide, and typically 25 μm thick, chuck-to-chuck length: 15 mm) in the glassy temperature range (*T* < *T*_g_) in the *XY* direction were measured by thermomechanical analysis (TMA) as an average in the range of 100–200 °C on a thermomechanical analyzer (Rigaku, Tokyo, Japan, TMA8311) with a fixed load (0.5 g per unit film thickness in μm, i.e., 12.5 g load for 25 μm thick films). Before the measurements, the first run was conducted to remove the adsorbed water of the specimens by heating up to 150 °C in the TMA chamber while flowing dry N_2_. After the sample was cooled to room temperature in the chamber, the specimen length was reset to 0% elongation, and the second heating run was conducted at a heating rate of 5 °C min^−1^ under a dry nitrogen flow (500 mL min^−1^) for the data collection.

#### 2.2.4. Heat Resistance

The storage modulus (*E’*), loss modulus (*E”*), and tan *δ* of the PEsI films (specimen size: 5 mm wide, 45 mm long, and typically 25 μm thick, chuck-to-chuck length: 25 mm) were measured in a nitrogen atmosphere by dynamic mechanical analysis (DMA, TA Instruments Japan, Tokyo, Japan, DMA-Q800). The measurements were conducted in an N_2_ atmosphere at a heating rate of 5 °C min^−1^ under a sinusoidal strain frequency of 0.1 Hz (amplitude: 0.1%) in the temperature range of 30–450 °C. The *T*_g_s (*α*-transition temperatures) and, if present, sub-*T*_g_s (*β*-transition temperatures: *T*_β_) for the PEsI films were determined from the peak temperatures in the *E”* curve, unless otherwise noted. The typical DMA curves are shown in Figures S1 and S2a. When the *β*-transitions observed around 160–200 °C in the DMA curve were weak or not distinct, they did not significantly contribute to an increase in the CTE, as suggested from a typical TMA curve [Figure S2b], where *T*_β_ is virtually undetected. This corresponds to the fact that the CTE values determined as the average in the range of 100–200 °C (our usual method) were not significantly different from the average of 100–150 °C below the *T*_β_.

The *T*_g_ values were also determined from the inflection point in the TMA curve, representing the point at which the specimens started to elongate abruptly, as the intersection of two tangential lines, as shown in Figure S2b. The TMA-based *T*_g_s were essentially equivalent to the DMA-based values, although the former tended to be 10–30 °C higher than the latter. TMA was useful in differentiating *T*_g_ from *T*_β_ based on the fact that the elongation at *T*_g_ is clearly significant in the TMA curves, compared to that at *T*_β_.

The thermal and thermo-oxidative stability of the PEsI films were evaluated using the 5% weight loss temperatures (*T*_d_^5^) determined by thermogravimetric analysis (TGA) on a thermo-balance (Netzsch Japan, Yokohama, Japan, TG-DTA2000S). TGA was conducted at a heating rate of 10 °C min^−1^ in a dry nitrogen and/or air atmosphere. A small weight loss due to water desorption at ~100 °C during the heating runs was compensated by off-setting at 150 °C to 0% weight loss for data analysis.

#### 2.2.5. Mechanical Properties

The tensile modulus (*E*), tensile strength (*σ*_b_), and elongation at break (*ε*_b_) of the PEsI films (specimen size: 30 mm long, 3 mm wide, and typically 25 μm thick, chuck-to-chuck length: 20 mm, valid specimen number: *n* = 10–20) were measured on a mechanical testing machine (A & D, Tokyo, Japan, Tensilon UTM-II) at a cross head speed of 8 mm min^−1^ at room temperature. The raw data were analyzed using a data processing program (Softbrain, Tokyo, Japan, UtpsAcS Ver. 4.09).

#### 2.2.6. Water Uptake

The water uptake (*W*_A_) for the PEsI films were determined according to the JIS K 7209 [28] standard using Equation (1):

*W*_A_ = [(*W*−*W*_0_)/*W*_0_] × 100 (1)

where *W*_0_ is the weight of a vacuum-dried film sample at 50 °C for 24 h, and *W* is the weight of the film immersed in water at 23 °C for 24 h and carefully blotted dry with tissue paper. A 50 μm thick KAPTON^®^ H film was used as a reference sample (*W*_A_ = 2.7% [16]) in each measurement of the specimens. Prior to each weighing, the static electricity of the specimens was discharged using a static elimination gun (Milty, Zerostat 3).

To investigate the influence of the imide group (O=C–N–C=O) in the PEsI structures on the *W*_A_, the contents of imide groups (*C*_i_, wt%) were calculated using Equation (2):

*C*_i_ = [*F*_W_ (imide)/*F*_W_ (unit)] × 100 (2)

where *F*_W_ (imide) and *F*_W_ (unit) denote the formula weights of the imide group and the repeating units of the PEsIs, respectively.

#### 2.2.7. Dielectric Properties

The dielectric constants (*ε*_r_ or DK) and dissipation factors (tan *δ* or DF) in the *XY* direction for the PEsI films were measured at a frequency of 10 GHz at 50% RH and 23 °C using a cavity resonator perturbation technique (IEC 62810) using a PNA-L network analyzer (Agilent Technologies, Tokyo, Japan, N5230A) and a cavity resonator (Kanto Electronic Application and Development Inc., Tokyo, Japan, CP531). Prior to the measurements, the specimens were exposed and stored at 50% RH and 23 °C for 24 h. The measurements were conducted by DJK Corp. (Yokohama, Japan) as a contracted service. The data were calibrated with a reference sample [polytetrafluoroethylene (PTFE), *ε*_r_ = 2.01, tan *δ* = 0.00023].

#### 2.2.8. Birefringence and Haze

The in-plane (*n*_in_ or *n*_xy_) and out-of-plane (*n*_out_ or *n*_z_) refractive indices of the PEsI films were measured at 589.3 nm (sodium lamp, *D*-line) on an Abbe refractometer (Atago, Tokyo, Japan, 4T, *n*_D_ range: 1.47–1.87) equipped with a polarizer by using a contact liquid (sulfur-saturated methylene iodide *n*_D_ = 1.78–1.80) and a test piece (*n*_D_ = 1.92). The thickness-direction birefringence (Δ*n*_th_) of the PEsI films, which represents the relative extent of chain alignment in the *XY* direction, was calculated from the following relationship:

Δ*n*_th_ = *n*_in_ − *n*_out_ (3)

The total light transmittance (*T*_tot_, JIS K 7361-1) and the diffuse transmittance (*T*_diff_, JIS K 7136) of the PEsI films were measured on a double-beam haze meter equipped with an integrating sphere (Nippon Denshoku Industries, Tokyo Japan, NDH 4000) to calculate the haze (turbidity) from the relationship:

Haze = (*T*_diff_/*T*_tot_) × 100 (4)

## 3. Results and Discussion

### 3.1. Difficulty in Simultaneously Achieving Low CTE, Low W_A_, Good Ductility, and Low DF

Figure 1 illustrates the difficulty in simultaneously achieving a low CTE along with other properties required for the present purpose. In this figure, the required items positioned to the right indicate a more challenging trade-off with a low CTE. We previously attempted to overcome the severe trade-off between low CTE and low tensile modulus [23], as well as the trade-off between low CTE and high thermo-processability [29], and we proposed these strategies.

In this study, the desired dielectric films must not only exhibit a low CTE but also a high *T*_g_, low water uptake (*W*_A_), sufficient film toughness, and low DF (yellow-highlighted items). Achieving both low CTE and high toughness is particularly challenging owing to conflicting molecular design requirements; the former necessitates stiff and linear main-chain structures, whereas the latter involves flexible chain structures, typically with rotatable ether linkages, to ensure sufficient chain entanglement. For instance, a rod-like structure of PI derived from PMDA and *p*-phenylenediamine (*p*-PDA) results in a very brittle film with an extremely low CTE, while a lab-made PMDA/4,4′-ODA PI produces a highly tough film without a low CTE [30].

However, the simultaneous achievement of a low CTE and DF does not appear to be difficult, as no clear trade-off relationship is known between them. Nonetheless, common polymeric materials with low DF usually do not have a high *T*_g_ and low CTE [31]. This highlights the fundamental difficulty in achieving a low CTE and DF simultaneously, as discussed later.

In this study, we initially visit the principles of DF control and propose a strategy for achieving an ultra-low DF while maintaining a high *T*_g_ and low CTE.

### 3.2. Necessity of Reducing DF of Dielectric Films

As previously stated, in a specific FPC used in mobile communication equipment, it is indispensable to suppress the transmission loss (*α*_tl_) of electrical signals running through the copper circuits to support high-speed communication under the 5Gstandard. *α*_tl_ is expressed as the sum of the conductor loss (*α*_c_) in the copper circuits and the dielectric loss (*α*_d_) in the dielectric films:

*α*_tl_ = *α*_d_ + *α*_c_ (5)

The *α*_c_ occurs closer to the surface of the copper foil with an increase in the operating frequency (*f*) and intensifies with an increase in its surface roughness. To suppress *α*_c_, it has become preferable to use copper foils with a lower surface roughness. However, this tendency has led to insufficient adhesion between the copper layers and dielectric films. In this study, we did not address the adhesion issue; instead, we focused on the properties of the neat dielectric film materials themselves.

On the other hand, *α*_d_ [dB/inch] in the dielectric films is proportional to the 0.5 power of *ε*_r_ and the first power of tan *δ*:

*α*_d_ = 27.3 (*f*/*c*) *ε*_r_^0.5^ tan *δ* (6)

where *f* and *c* denote the operating frequency and the speed of light, respectively. In the range of *f* < 1 MHz, *α*_c_ usually predominates over *α*_d_. However, with increasing *f*, the contribution of *α*_d_ increases, making *α*_d_ predominant over *α*_c_ in the higher *f* range (>~1 GHz) [32]. According to Equation (6), to reduce tan *δ* and *ε*_r_, particularly tan *δ* with an exponent of the first power is effective in suppressing *α*_d_ in the GHz range.

*ε*_r_ corresponds to the electric charge-storing ability, and tan *δ*, which depends on *f* and temperature [33], represents the conversion efficiency from microwave energy to heat. Strictly speaking, these dielectric parameters of polymer films, in contrast to those of liquid samples, also depend on the measuring method, owing to their orientational anisotropy (if any). In this study, the dielectric parameters in the *XY* direction were evaluated under fixed conditions (10 GHz, 50% RH, and 25 °C).

### 3.3. Primary Factor for Controlling Dielectric Properties in the GHz Range

The strategies to reduce *ε*_r_ are currently wellestablished. *ε*_r_ is expressed by the ClausiusMossotti formula [34]:

*ε*_r_ = [1 + 2 Σ (*P*_m_/*V*_m_)]/[1 − Σ (*P*_m_/*V*_m_)] (7)

where *P*_m_ and *V*_m_ denote the molar polarization and molar volume of each group in the polymer structure, respectively. This equation indicates that the incorporation of groups with a lower *P*_m_/*V*_m_ ratio, typically CF_3_ groups, is effective in reducing *ε*_r_, as observed in actual CF_3_-containing PIs [10,35,36,37,38,39]. Another effective approach is to reduce the content of highly polarized imide groups (*C*_i_) using high-molecular-weight monomers, including less-polarized aromatic units [40]. Reducing π electrons using cycloaliphatic monomers also significantly lowers *ε*_r_ [41,42]. As mentioned earlier, the adsorbed/absorbed water deteriorate the dielectric properties owing to their high DK and DF values (*ε*_r_ = 80.4, tan *δ* = 0.123 at 2.45 GHz and 25 °C [43]). In a fixed PI system, these dielectric properties linearly deteriorate with an increase in the relative humidity as the measuring condition [44]. A relatively good correlation was observed between *W*_A_ and *C*_i_ [20,21,22], suggesting that controlling *C*_i_ is undoubtedly an effective approach for reducing *W*_A_. Based on the lowest *ε*_r_ of air (1.0), another effective approach to reduce *ε*_r_ is to form nano-pores in PI films [45]. Low-DK materials (typically PTFE) can be dispersed in dielectric films to reduce *ε*_r_. However, there are concerns regarding these composites; the required properties of PI films (film toughness, heat resistance, CTE, and haze) likely deteriorate significantly at high dispersoid contents. Therefore, we did not address these composite systems in this study.

However, the strategies for DF control are not always fully understood. There are various polarization phenomena over a wide *f* range [46]: (1) interfacial polarization typically observed in ceramic sintered moldings (*f* = ~1 Hz–1 kHz), (2) orientational polarization observed as a dielectric heating phenomenon (~3 MHz–30 GHz), (3) ionic polarization observed in ionic crystals (~3–30 THz), and (4) electronic polarization (~3–30 PHz). This study focuses on the orientational polarization, which occurs with the rotational and orientational motions of permanent dipoles induced by high-*f* electric fields. The slightly delayed orientational motion against the applied high-*f* electric fields contributes to an increase in DF. That is, the DF decreases when the rotational motions completely follow a low-*f* electric field or cannot follow a high-*f* electric field at all, and the DF becomes maximal at medium frequencies. For example, water (25 °C) shows an almost symmetric *log f*–DF curve with a single peak at about 20 GHz [27].

Unlike fluid materials such as water, in solid PI films without large molecular motions, to reduce their DF in the GHz range, it is unrealistic (impossible) to completely shift the rotational motions to the “electric field-following” type (ultra-high mobility type). Alternatively, the complete inhibition of local rotational motions (i.e., shift to “electric field-non-following” type) is applicable and effective in reducing the DF of solid PI films.

A prominent effect of hindering molecular motions on reducing DF is observed in the following typical example; ice (–12 °C) exhibits a dramatically decreased tan *δ* (0.0009 at 2.5 GHz [47]), whereas water (25 °C) has a very high tan *δ* (0.123 at 2.45 GHz [43]). A similar effect of hindering molecular motions is observed in the *log f*–DF curves at varying temperatures for water; water (50 °C) shows a monomodal *log f*–DF curve with a peak at *f*_max_ = 50 GHz. As the temperature decreases, a low-frequency shift of the curve occurs, and *f*_max_ reaches 10 GHz at 10 °C [27]. The observed low-frequency shift (i.e., expansion of the above-mentioned electric field-non-following region) very likely reflects the decreased rotational mobility of water molecules owing to the increased contribution of hydrogen bonding with decreasing temperature.

**Figure 6:**
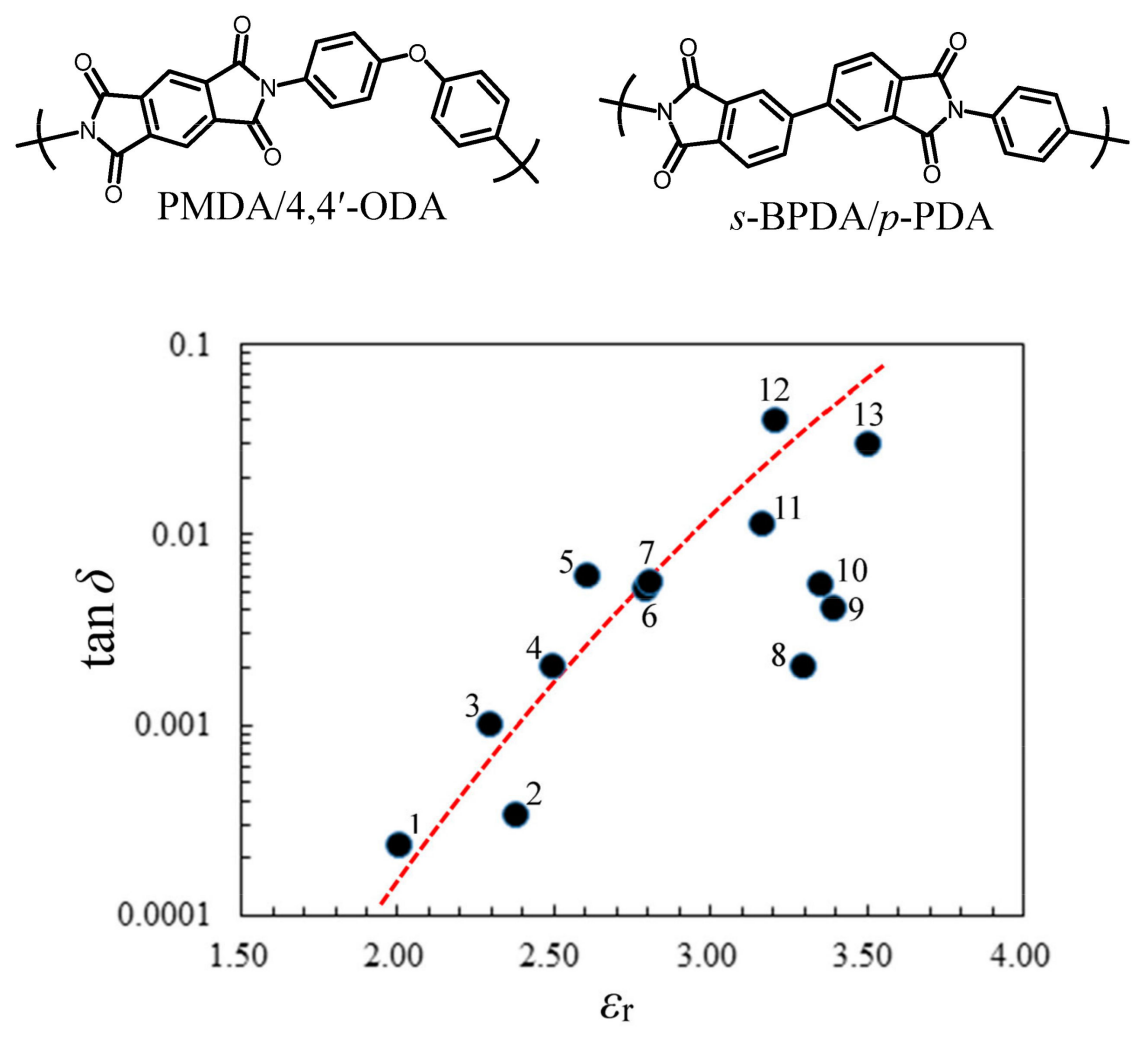
The correlation of *ε*_r_ with tan *δ* in the frequency range of 1–28 GHz for various polymeric materials: (1) PTFE, (2) COP, ZEONOR^®^, (3) PE, (4) PPE, (5) PMMA, (6) PVC, (7) PAr, (8) LCP, Vecstar^®^, (9) PES, (10) PI (*s*-BPDA/*p*-PDA), (11) PI (PMDA/4,4′-ODA), (12) PET, and (13) EP. The structures of conventional PIs are shown at the top of this figure.

In addition to the above-mentioned molecular mobility, the polarity control of structural units in polymers is important for suppressing DF. Figure 6 shows the relationship between *ε*_r_ and tan *δ* in the range of 1–20 GHz for common polymers and conventional PIs: (1) PTFE [48], (2) cyclo-olefin polymer (COP, ZEONOR^®^) [48], (3) polyethylene (PE) [49], (4) poly(2,6-dimethyl-1,4-phenylene ether) (PPE) [50], (5) poly(methyl methacrylate) (PMMA) [51], (6) poly(vinyl chloride) (PVC) [51,52], (7) polyarylate (PAr, bisphenol A/isophthaloyl chloride) [53], (8) liquid-crystalline polyester (LCP, Vecstar^®^) [54,55], (9) poly(ether sulfone) (PES) [56], (10) PI (*s*-BPDA/*p*-PDA), (11) PI (PMDA/4,4′-ODA), (12) poly(ethylene terephthalate) (PET) [57], and (13) epoxy resin (EP, bisphenol A diglycidyl ether/4,4′-diaminodiphenyl sulfone) [58]. A rough correlation is observed over the wide ranges of tan *δ* (0.0001–0.1) and *ε*_r_ (2–4). Specifically, non-polar polymers without highly polarized groups (–NHCO–, –NHCOO–, –NHCONH–, >C=O, –OH, etc.) such as PTFE, COPs, PE, and PPE exhibit quite low DF values. Thus, the primary strategy for suppressing DF is undoubtedly to remove or reduce the highly polarized units from the polymer structures. However, in fact, these non-polar polymers have neither high *T*_g_ nor low CTE [31], e.g., *T*_g_ = 140 °C for ZEONEX^®^ (COP) [48] and 226 °C for PPE [50]. This again suggests the difficulty in achieving the goal of this study.

### 3.4. Connecting Groups Effective in Simultaneously Reducing CTE and DF

**Figure 7:**
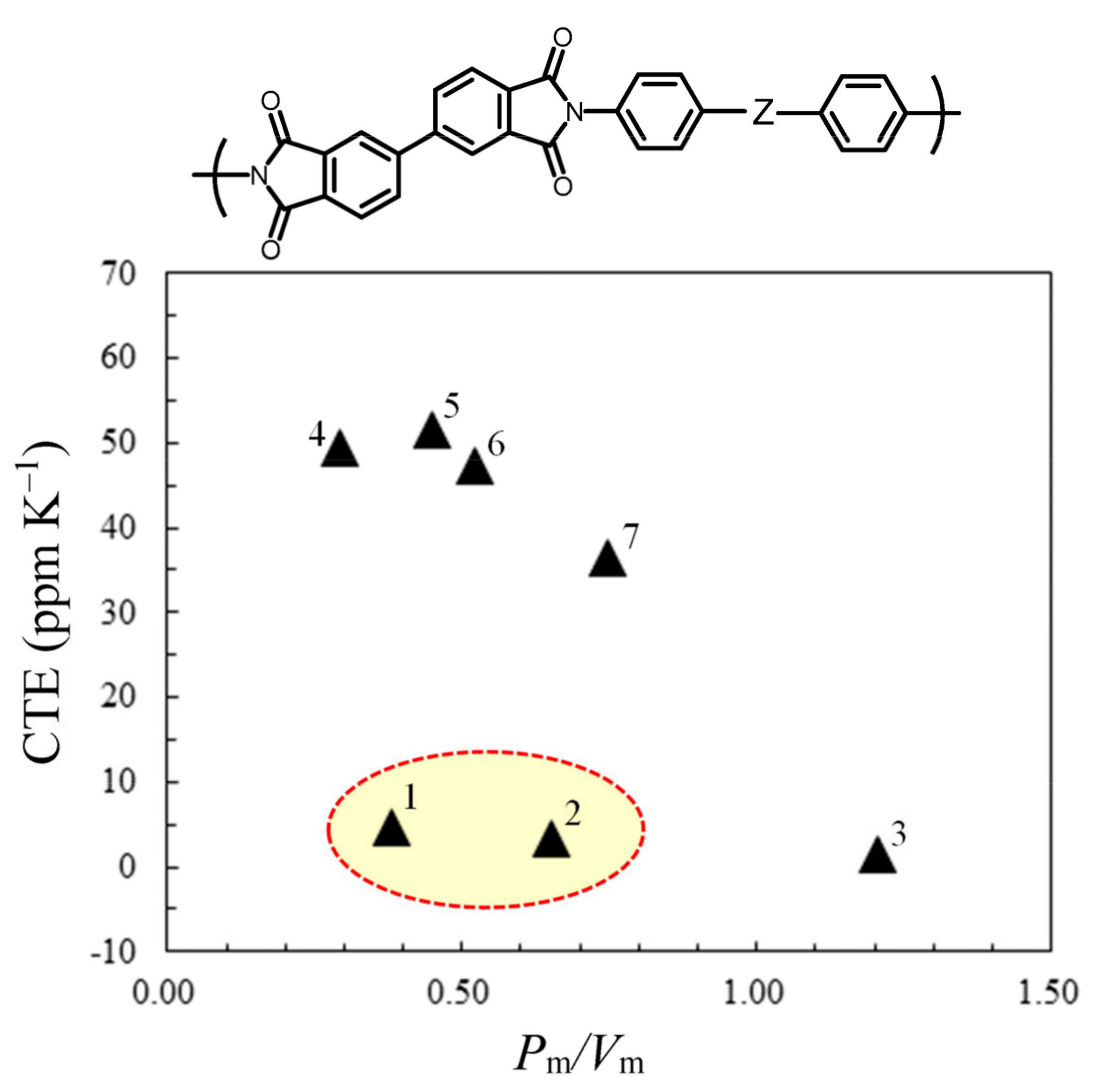
CTE plotted against the *P*_m_/*V*_m_ ratio of the connecting groups (Z) used in the diamines for PI systems obtained from *s*-BPDA and diamines with different *Z* groups: (1) *para*-phenylene, (2) –O–CO–, (3) –NH–CO–, (4) –CH_2_–, (5) –S–, (6) –O–, and (7) >C=O groups. The plots surrounded by a broken line represent *Z* groups advantageous for obtaining low-CTE and low-DF PIs. The structures of the focused PIs are shown at the top of this figure.

The CTE of PI films strongly depends on chain linearity, which is affected by the connecting groups incorporated in the monomers used [59]. Figure 7 shows the CTE values plotted against the *P*_m_/*V*_m_ ratios [34] of the connecting groups (*Z*) for the PIs derived from *s*-BPDA and *Z*-containing diamines. The methylene (plot #4, *P*_m_/*V*_m_ = 0.293) and ether (plot #6, *P*_m_/*V*_m_ = 0.520) groups with low *P*_m_/*V*_m_ values are the connecting groups suitable for reducing DF. However, these connecting groups decrease the main-chain linearity and consequently cause an increase in the CTE. In contrast, the amide connecting group (plot #3) has a significant effect on reducing the CTE; however, its high *P*_m_/*V*_m_ value (1.205) is disadvantageous for reducing the DF.

On the other hand, the *para*-phenylene connecting group (plot #1), which has a very low *P*_m_/*V*_m_ value (0.382), results in a very low CTE. Thus, the *para*-phenylene group is optimal as the connecting group for the present purpose. However, the synthesis of certain monomers including the 4,4″-*p*-terphenylene (TP) unit involves quite complicated processes such as the cross-coupling reaction [60]. Instead of the TP unit, the introduction of the 4,4′-biphenylene unit into PI backbones is more realistic and practical because the related raw materials, such as 44BP and HPBA, are readily available.

On the other hand, the ester connecting group is superior to the *para*-phenylene group in terms of the simplicity of monomer synthesis. In addition, the former (plot #2) resulted in a low CTE and has a relatively low *P*_m_/*V*_m_ value (0.652), which was approximately half of that of the amide group (1.205). These results suggest that the use of specific monomers, including multiple 4,4′-biphenylene units *para*-linked via ester groups, can be an optimal approach to achieve low CTE and low DF simultaneously.

### 3.5. Problems Arising from Longitudinal Structural Extension in Ester-Linked Monomers

**Figure 8:**
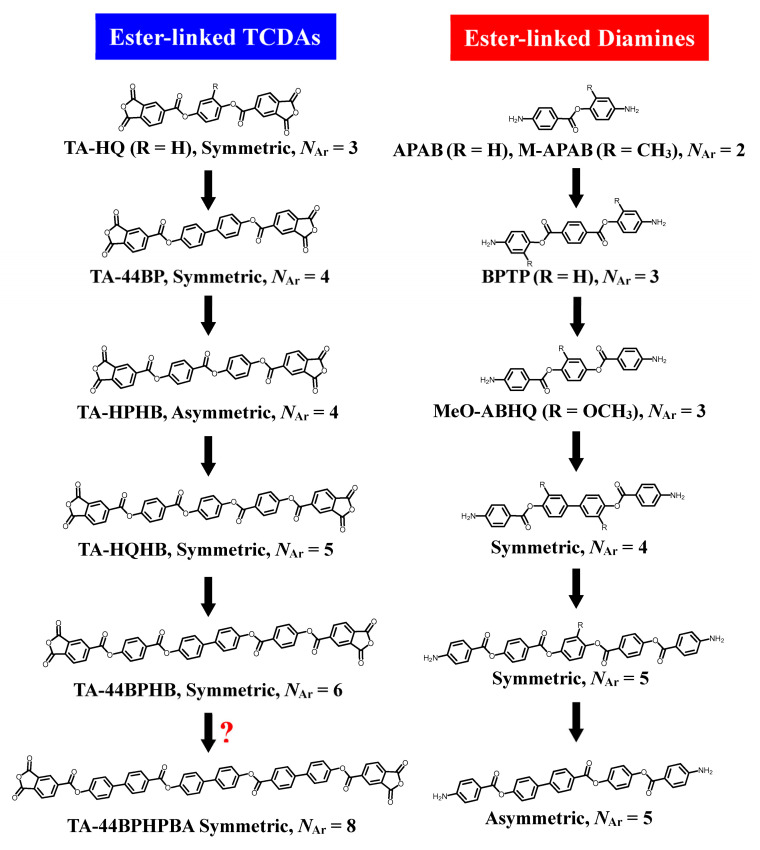
The transition of monomers that we have handled to obtain low-*W*_A_ and low-CTE high-temperature polymers. The question mark represents that it is unclear whether the product can be obtained.

Figure 8 shows the transition of our ester-linked monomers for obtaining heat-resistant polymers with a low *W*_A_ and low CTE. However, these ester-linked diamines involved a serious problem; their longitudinal structural extension (aromatic ring number; *N*_Ar_ ≥ 4) led to a significant decrease in the solubility in NMP at room temperature. Consequently, the common equimolar polyaddition procedure, where solid TCDA is added to a homogeneous NMP solution of diamine at room temperature, became difficult to apply [22]. In some cases, even poorly soluble diamines can barely be dissolved in NMP by heating. However, it is undesirable to add solid TCDA to a hot NMP solution because this procedure often results in a significant decrease in the molecular weight of the finally obtained PAA. When the diamines are completely insoluble even in hot NMP, the reaction with TCDAs does not proceed at all in many cases [23].

In contrast, even if the TCDAs were almost insoluble in NMP at room temperature, it was not as severe compared to the solubility problem of diamines. This is because the solid TCDAs added to the diamine solution only need to be slightly dissolved, and the reaction can continue while gradually dissolving the initially undissolved portion of TCDA. Therefore, in this study, we applied an approach to increase the *N*_Ar_ to TCDAs without severe solubility restrictions. In our previous studies, even when an ester-linked TCDA with *N*_Ar_ = 6 was used, polyaddition with common aromatic diamines proceeded smoothly [21]. However, in the present study, there were concerns caused by the further increase in the *N*_Ar_, as shown in Table 3.

**Figure 9:**
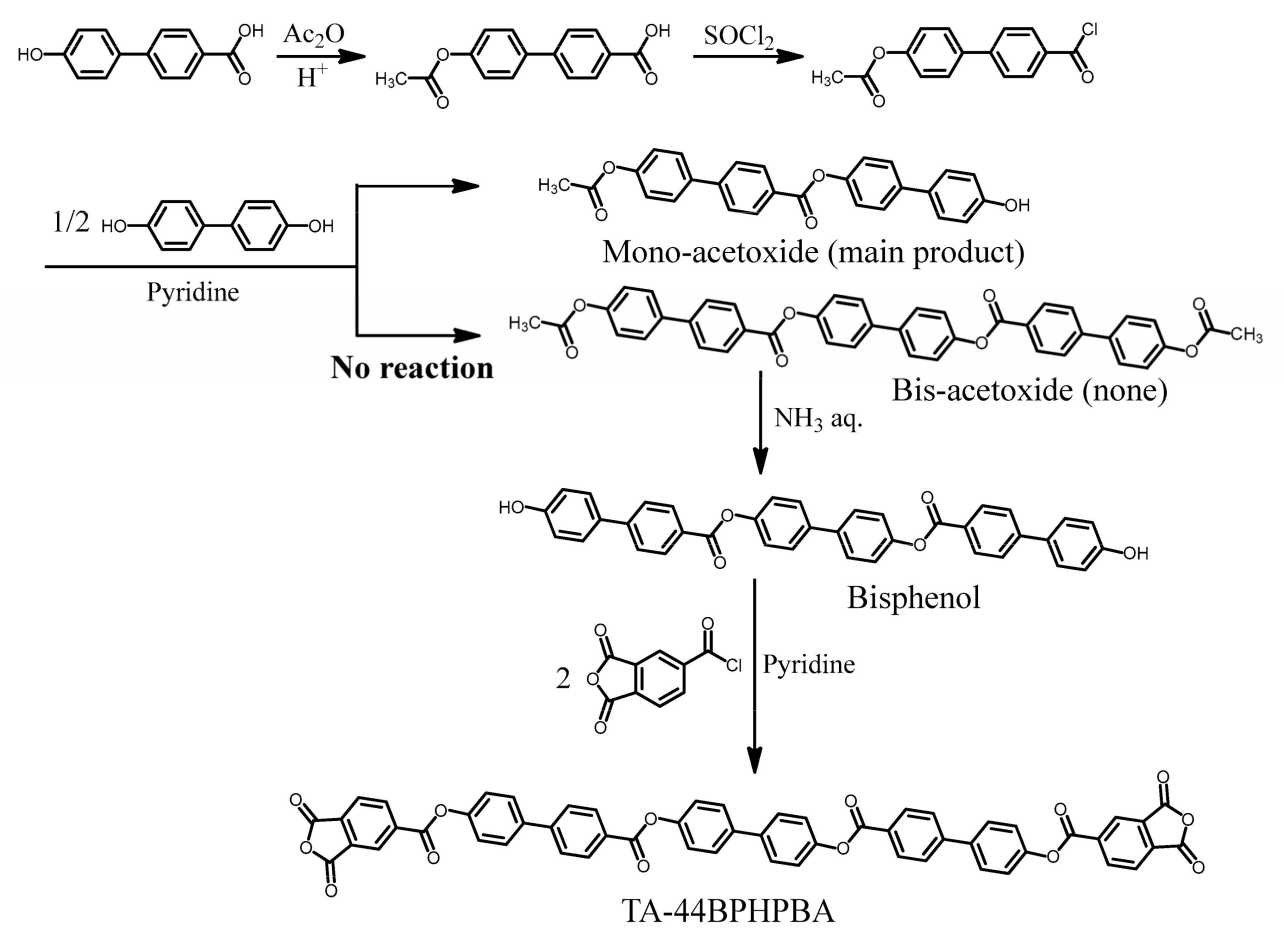
A problem during the synthesis of a new ester-linked TCDA with a further longitudinally extended structures containing three *p*-biphenylene units (*N*_Ar_ = 8).

Based on the considerations in Section 3.4, we initially attempted to synthesize a new TCDA [TA-44BPHPBA (*N*_Ar_ = 8), Figure 8, lower left], in which multiple 4,4′-biphenylene units were all-*para*-linked via ester groups, according to the reaction scheme shown in Figure 9. However, it was difficult to obtain the desired intermediate (bis-acetoxide) owing to the precipitation of the insoluble mono-acetoxide. Thus, our initial attempt failed during the middle of the monomer synthesis, as indicated in the concern shown in Table 3(1).

A possible solution to this problem was to introduce some bulky substituent to the central 4,4′-biphenylene unit. Our initial attempt was to use TFMB as a starting material for the new TCDA synthesis, with the expectation of the solubility-improving effect of its CF_3_ substituents. Consequently, a TCDA (TA-TFMBHPBA, Scheme 8) was successfully obtained without experiencing the precipitation of the undesirable mono-acetoxide. Although this TCDA contains amide groups, which are likely undesirable for DF control, there is a chance that their negative effect is almost canceled out or diluted by the presence of two adjacent CF_3_ groups.

**Figure 10:**
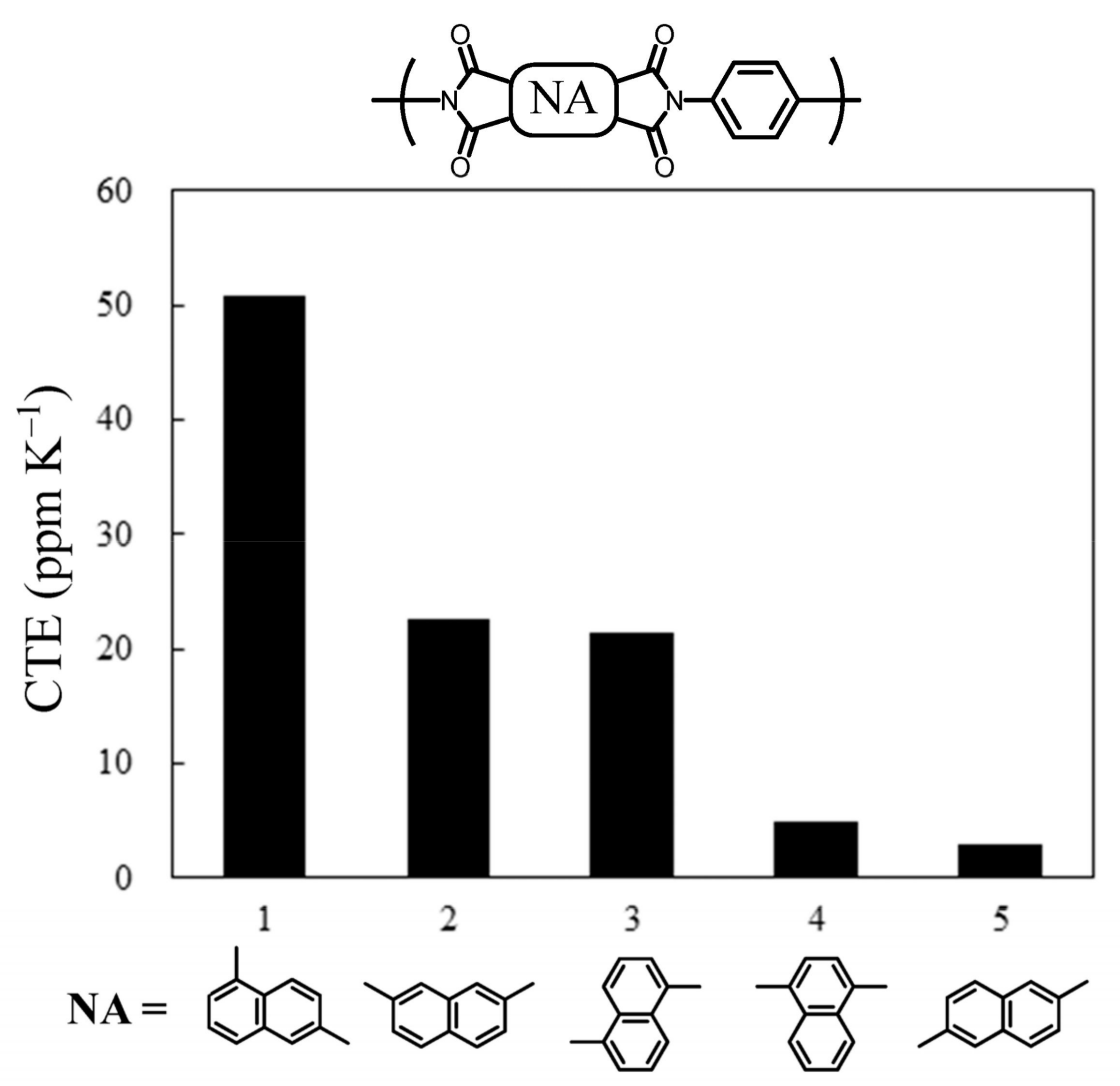
The CTEs of the PEsI films obtained using *p*-PDA and TCDAs consisting of dihydroxynaphthalene (DHNA) isomers: (1) 1,6-, (2) 2,7-, (3) 1,5-, (4) 1,4-, and (5) 2,6-DHNA.

Another idea was to introduce naphthalene units instead of the 4,4′-biphenylene unit. The slightly bulkier structure of the naphthalene units was expected to improve the solubility of the intermediates during the synthesis of the TCDAs. We previously studied the properties of PEsIs obtained from simple ester-linked TCDAs using various isomeric dihydroxynaphthalenes (DHNAs) [61]. A part of the results is shown in Figure 10. A significant isomer effect of the DHNAs on the CTE was observed; only when using 1,4- and 2,6-DHNA, which lead to TCDAs with highly linear structures, low-CTE PEsI films were obtained. From the results, in the present study, we decided to synthesize new longitudinally extended TCDAs, including the 2,6-naphthalene (26NA) central unit, instead of the 4,4′-biphenylene unit. This choice was made due to the easier availability of 26NA-based raw materials compared to 14-NA-based ones. Consequently, the 26NA-containing TCDAs [TA-26NAHNA (*N*_Ar_ = 8), Scheme 5], as well as the other related TCDAs (*N*_Ar_ = 6 and 7), were successfully synthesized without any solubility problems, as mentioned in the Experimental Section. Thus, the concern listed in Table 3(1) was dispelled.

Other serious concerns arose from the *N*_Ar_-increasing approach; namely, the inhibition of polyaddition due to the complete insolubility of the new TCDAs [Table 3(2)] and the gelation or precipitation of the PAA solutions during polyaddition [Table 3(3)]. It is accepted that hydrogen bonding of the COOH and NHCO groups in PAAs with NMP [62,63] supports the solvation of PAAs in NMP. This suggests that gelation or precipitation during polyaddition likely occurs owing to the significantly decreased content of these solvating groups resulting from the *N*_Ar_-increasing approach [Table 3, bottom, (a)].

### 3.6. Polymerizability of Longitudinally Extended Structures of Ester-linked TCDAs and Properties of PEsI films

#### 3.6.1. TA-TFMBHPBA-Based Systems (*N*_Ar_ = 8)

The results of the polyaddition of TA-TFMBHPBA with common aromatic diamines (*p*-PDA and 4,4′-ODA) and the properties of the resulting PEsI films are summarized in Table 4. This TCDA showed high polyaddition reactivity with these diamines and led to homogeneous and viscous solutions of PAAs with high *η*_red_ values (>2 dL g^−1^). Thus, the concerns listed in Table 3(2) and (3) were dispelled in the TA-TFMBHPBA-based systems. The PAA cast films were hazeless. Thermal imidization of the PAA films produced a ductile PEsI film that did not fracture in a simple bending test at a zero-curvature radius (*r*_c_).

The system using *p*-PDA as the diamine (sample #1) afforded a hazy PEsI film (haze = 69.6%), as shown in Figure S3a. The DMA curve showed an extremely high *T*_g_ (395 °C) with a *T*_β_ (200 °C). This PEsI film exhibited a high tensile modulus (5.76 GPa) and relatively low CTE (27.5 ppm K^−1^), reflecting its stiff and linear chain structure. This system (#1) contains thermally less stable amide and ester groups than commonly used ether groups [59]. Therefore, its thermal stability [*T*_d_^5^ (N_2_) = 476 °C] was unavoidably inferior to those of wholly aromatic PIs (520–570 °C, measured at 10 °C min^−1^ [59]), although it still maintains a sufficiently high thermal stability. In addition, despite the presence of polar amide groups in its structure, this PEsI (#1) exhibited a much lower water uptake (*W*_A_ = 0.70%) than that of a home-made PMDA/4,4′-ODA PI film (*W*_A_ = 2.3–2.5%) and a PMDA/4,4′-diaminobenzanilide (DABA) film (3.4%). This likely reflects that the hydrophilicity of the amide groups was “diluted” by a hydrophobic effect of the adjacent CF_3_ groups. This system (#1) also had a low tan *δ* (0.00776) compared to that of a home-made PMDA/4,4′-ODA PI film (0.0114 at 10 GHz); however, it remained much higher than a temporary target DF (0.002 at 25 GHz for Vecstar^®^ film [54,55]). This suggests that the negative effect of the amide groups on the DF was not easily canceled out by the adjacent CF_3_ groups, unlike the relatively significant *W*_A_-reducing effect of the CF_3_ groups.

When using 4,4′-ODA (#2) instead of *p*-PDA, we observed a certain improvement in *ε*_b_, although the effect was not as pronounced as expected. Despite the use of 4,4′-ODA containing a flexible ether group, this system (#2) showed a high *T*_g_ (377 °C by DMA) with a *T*_β_ (199 °C) and a relatively low CTE (33.4 ppm K^−1^). These results probably reflect the combined effects of very high chain rigidity based on the all-*para*-linked TCDA-based diimide (TCDI) units and interchain hydrogen bonding between the amide groups.

#### 3.6.2. TA-26NAHB-Based PEsI Systems (*N*_Ar_ = 6)

We previously reported excellent dielectric properties at 10 GHz for a PEsI film obtained using an ester-linked TCDA with *N*_Ar_ = 6 (TA-44BPHB, Table 5, bottom) [21]. In the present study, to investigate the effect of the 26NA unit, we synthesized TA-26NAHB, in which the central 4,4′-biphenylene unit of TA-44BPHB was replaced with the 26NA unit. Table 5 summarizes the results of polyaddition with common diamines (*p*-PDA, 4,4′-ODA) and the properties of the PEsI films. TA-26NAHB showed high polyaddition reactivity with these diamines and led to homogeneous and viscous solutions of PAAs with sufficiently high *η*_red_ values (molecular weights). Thus, there were no concerns listed in Table 3(2) and (3). Thermal imidization of the hazeless PAA cast films afforded ductile films resistant to the zero-*r*_c_ bending tests, except for the TA-26NAHB/*p*-PDA system (#3).

The *p*-PDA system (#3), in contrast to highly hazy LCP films, afforded a visually completely clear PEsI film (haze = 3.9%), as shown in Figure S3b. This characteristic is favorable for the high-precision positioning of electronic parts through the films (alignment process). However, this PEsI film proved to be brittle, making it difficult to conduct the mechanical stretching test properly. In general, the film toughness for linear polymers, as well as PI systems, is significantly influenced by their main chain structures when their molecular weights are sufficiently high [30,64]. Therefore, the brittleness of this film (#3) is probably ascribed to poor chain entanglement based on its highly linear chain structure. The rigid chains also contributed to a very high *T*_g_ [363 °C by DMA (Figure S1)] and ultra-low CTE (5.1 ppm K^−1^). The latter results from a high extent of chain alignment in the *XY* direction (in-plane orientation), which is significantly enhanced during thermal imidization of PAA cast films fixed on the substrates [65,66,67,68,69]. The in-plane orientation of PIs (particularly, PMDA/4,4′-ODA film) was observed using various techniques [70,71,72,73,74].

It is noteworthy that high-quality PEsI films, similarly to conventional PIs, were easily obtained through a simple process (thermal imidization of the PAA cast films), in contrast to LCP films, which often require homogeneous stretching in the *XY* direction by inflation processing to improve the tearability intrinsic to LCPs and low-CTE properties [54,55]. Additionally, the PEsI film (#3) achieved a relatively low water uptake (0.52%) and an extremely low DF (0.00267), even though the DK was at a common level (3.22). Thus, in this system, the DF-reducing effect observed was more pronounced than the *W*_A_-reducing effect.

The PEsI system (#3) was compared with a related PEsI derived from a simple 26NA-containing TCDA (TA-26NA, Table 5, bottom) and *p*-PDA. The latter had a much higher DF (0.00501) and *W*_A_ (1.03%) than the TA-26NAHB/*p*-PDA system (#3), as shown in Table 5. Thus, this simple TCDA (TA-26NA) was much less effective in reducing DF and *W*_A_ than the longitudinally extended TA-26NAHB.

The use of 4,4′-ODA (#4) instead of *p*-PDA afforded a very hazy PEsI film (haze = 87.1%), as shown in Figure S3c, with a somewhat improved *ε*_b_. A significantly decreased *T*_g_ (250 °C) with the appearance of a distinct *T*_β_ (163 °C), in addition to a significantly increased CTE (55.0 ppm K^−1^), was observed probably owing to the increased rotational flexibility originating from 4,4′-ODA. On the other hand, DK (3.01) and DF (0.00206) were discernibly reduced. The clearly reduced DF is probably ascribed to a positive contribution of the biphenyl ether units in the TA-26NAHB/4,4′-ODA, corresponding to the fact that PPE has a very low DF value (0.002 at 1 GHz, Figure 6). Thus, in the 4,4′-ODA system (#4), neither the decreased *T*_g_ nor the generation of the distinct *T*_β_ exerted any direct adverse influence on the DF measured at room temperature. This is probably attributed to the quite different modes of these molecular motions; *T*_g_ and *T*_β_ reflect the micro-Brownian motions over the entire main chains and local cooperative motions, respectively, whereas DF at room temperature is associated with local and much smaller rotational motions (wagging motion) in the glassy solid films.

4,4′-ODA was copolymerized with a minor content (25 mol%) to improve the brittleness of the TA-26NAHB/*p*-PDA system (#3). The resulting copolymer (#5) afforded a visually completely clear PEsI film (haze = 2.6%), as shown in Figure S3d, with somewhat improved toughness. This PEsI film maintained an extremely low CTE (10.5 ppm K^−1^) and a very high *T*_g_ (362 °C) with a weak *β*-transition (174 °C) in the DMA curve [Figure S2a], which was virtually undetected by TMA [Figure S2b]. Whereas the water uptake (0.88%) of this copolymer was not as low as expected, a more pronounced DF-reducing effect was observed (tan *δ* = 0.00191 at 10 GHz, Table 5), which is comparable to or lower than the DF of an LCP (0.0022 at 25 GHz for Vecstar^®^ film [54,55]). Thus, a feature common to the TA-26NAHB-based systems was confirmed again, viz., the 26NA unit incorporated into the central bisphenol unit had a remarkable effect on suppressing DF, compared to reducing *W*_A_, as shown at the bottom of Table 5.

The 26NA-containing copolymer (#5) was also compared with the related copolymer, the TA-44BPHB/*p*-PDA(75);4,4′-ODA(25) system with a different central unit in the TCDA (4,4′-biphenylene unit). The copolymer system (#5) was superior to its counterpart in terms of DF (0.00191 for the former and 0.00276 [21] for the latter). This is difficult to explain by the difference in the imide group contents (*C*_i_) of these systems; the former has a slightly higher *C*_i_ (16.60 wt%) than the latter (16.15 wt%). An alternative explanation is that the high *f*-induced local rotational mobility of the central 26NA unit in the former was more restricted than that of the central 4,4′-biphenylene unit in the latter.

#### 3.6.3. Local Motions of Permanent Dipoles against High-*f* Electric Fields

**Figure 11:**
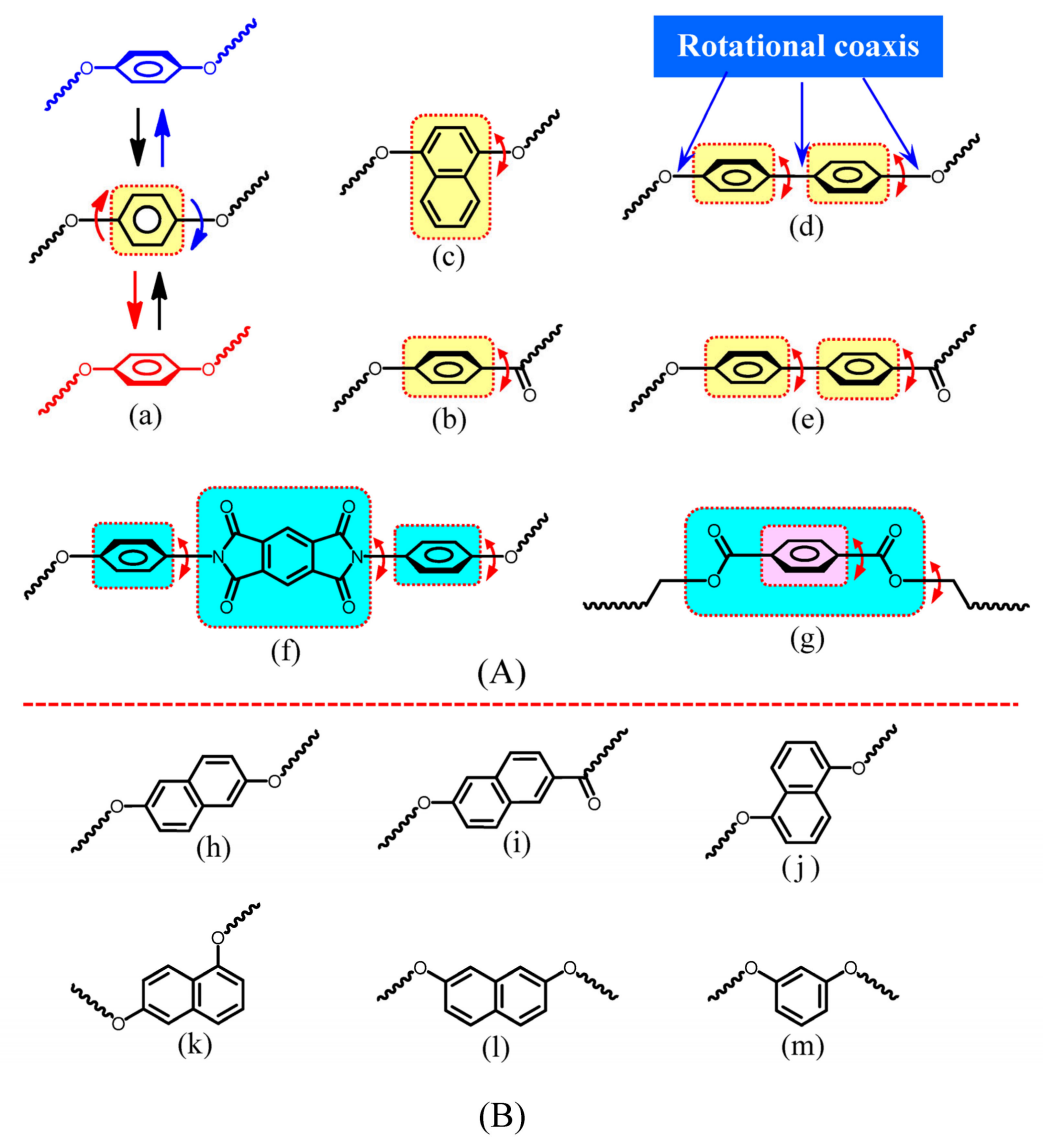
Structural units with (**A**) and without rotational coaxis (**B**) in the main chains. (**A**): (**a**) HQ, (**b**) HBA, (**c**) 14DHNA, (**d**) 44BP, (**e**) HPBA units in the PEsIs, (**f**) *N*,*N*-diphenylpyromellitimide unit in PMDA/4,4′-ODA, (**g**) diethyl terephthalate unit in PET; and (**B**): (**h**) 26DHNA, (**i**) HNA, (**j**) 15DHNA, (**k**) 16DHNA, (**l**) 27DHNA, and (**m**) resorcinol units.

Figure 11 compares the local rotational mobility, which contributes to an increase in DF, for various structural units incorporated into the PI or PEsI main chains. For example, in a TCDI unit containing a central hydroquinone (HQ) unit, the small rotational motion (wagging motion) of the 1,4-phenylene unit can be allowed around its rotational coaxis in response to high-*f* electric fields [Figure 11A(a)]. However, its wagging motion must be quite limited owing to the surrounding frozen molecules in the glassy solid films. Therefore, in addition to the presence of the rotational coaxis, a free volume around the 1,4-phemylene unit is indispensable for its wagging motion in solid films. In addition to the HQ unit (a), HBA (b), 14DHNA (c), 44BP (d), HPBA (e), the pyromellitimide (PMDI) unit, and the adjacent *N*-phenylene unit (f) also have similar rotational coaxes. In particular, the PMDI unit containing four polarized C=O groups is very likely to increase the DF. Indeed, a home-made PMDA/4,4′-ODA PI film showed a very high DF (0.0114 at 10 GHz). Similarly, when the terephthalate units in poly(ethylene terephthalate) (PET) adopted a specific conformation shown in Figure 11A(g), a rotational coaxis is generated; consequently, the wagging motion of the terephthalate unit can be allowed. This corresponds to an exceedingly high DF of the PET film (0.039 at 1 GHz [57]).

In contrast, the rotational mobility of the 26DHNA unit [Figure 11B(h)] and the HNA unit (i) is probably considerably restricted owing to the absence of the rotational coaxis and the ease of inter-stacking due to their planar structures. This is a possible reason why the TA-26NAHB-based PEsIs exhibited the pronounced DF-reducing effect. Similarly, the other isomeric naphthalene units [Figure 11B(j)–(l)] and the *meta*-phenylene unit (m) have no rotational coaxis (suitable for reducing DF), although their non-linear structures disturb the generation of low CTE (Figure 10). This tendency, except for the 26NA unit, suggests that there is indeed a trade-off relationship between low DF and low CTE (Figure 1).

#### 3.6.4. TA-HQHNA-Based PEsI Systems (*N*_Ar_ = 7)

Table 6 summarizes the results of the polyaddition of TA-HQHNA, which contains two 26NA units, with common aromatic diamines (*p*-PDA and 4,4′-ODA) and the properties of TA-HQHNA-based PEsI films. This TCDA showed high polyaddition reactivity with these diamines and led to homogeneous and viscous solutions of PAAs with sufficiently high molecular weights. Thus, there were no concerns listed in Table 3(2) and (3). The PAA cast films were hazeless. The thermally imidized films were ductile, with resistance to the zero-*r*_c_ bending tests.

The *p*-PDA system (#6) afforded a visually quite clear PEsI film (haze = 13.7%), as shown in Figure S3e. This film, as well as its counterpart (#3), exhibited a considerably high *T*_g_ (373 °C) and ultra-low CTE (1.9 ppm K^−1^), which corresponds to a very high thickness-direction birefringence (Δ*n*_th_ = 0.132) that represents a high extent of in-plane chain orientation. Although the DF-reducing effect was not as pronounced as that of its counterpart (#3), as listed in Table 6, the *W*_A_ was dramatically reduced (0.21%).

The use of 4,4′-ODA (#7) instead of *p*-PDA produced an almost clear PEsI film (haze = 9.6%), as shown in Figure S3f, with a somewhat improved toughness. Despite the presence of flexible ether linkages in its main chains, this film maintained a very high *T*_g_ (331 °C) with a *T*_β_ (178 °C) and a relatively low CTE (27.2 ppm K^−1^). In addition, we observed a significant DF-reducing effect by replacing *p*-PDA with 4,4′-ODA (DF = 0.00347 → 0.00189), as similarly seen in their TA-26NAHB-based counterparts (DF = 0.00267 → 0.00206). These results also reflect the afore-mentioned PPE-like biphenyl ether effect.

In the 4,4′-ODA system (#7), a prominent *W*_A_-reducing effect (0.21%) was observed, as in the *p*-PDA system (#6). This feature (Table 6, bottom) can be explained as follows: the planar and hydrophobic 26NA units, which are connected near the water-attracting benzimide (BI) units, can assist the inter-stacking between the BI units, consequently reducing the water-entering nano-spaces in the vicinity of the BI units.

#### 3.6.5. TA-26NAHNA-Based PEsI Systems (*N*_Ar_ = 8)

To ascertain the features of the TA-26NAHB (DF-reducing effect, Table 5) and TA-HQHNA (*W*_A_-reducing effect, Table 6) systems, a combined type of TCDA (TA-26NAHNA, *N*_Ar_ = 8) was synthesized. If each feature of them is true, the TA-26NAHNA-based PEsIs are expected to achieve an extremely low DF and *W*_A_ simultaneously.

Table 7 summarizes the results of the polyaddition using TA-26NAHNA and the properties of TA-26NAHNA-based PEsIs. This TCDA showed high polyaddition reactivity with *p*-PDA, 4,4′-ODA, APAB, and M-APAB and led to homogeneous solutions of PAAs with sufficiently high *η*_red_ values. Thus, the concerns listed in Table 3(2) and (3) were dispelled despite the significant structural extension of TCDA to *N*_Ar_ = 8. The PAA cast films were hazeless. The thermally imidized TA-26NAHNA-based PEsI films were ductile, as suggested by their resistance to the zero-*r*_c_ bending tests.

The TA-26NAHNA/*p*-PDA system (#8) afforded almost clear PEsI films (haze = 15.1%). This PEsI film, as well as its *p*-PDA-derived counterparts (#3 and #6), achieved an extremely high *T*_g_ (371 °C), ultra-low CTE (3.8 ppm K^−1^), and very high tensile modulus (7.70 GPa), reflecting its main chain stiffness/linearity. The observed ultra-low CTE and high modulus are ascribed to significant in-plane chain orientation induced by thermal imidization, corresponding to a very high Δ*n*_th_ value (0.129). However, the *ε*_b_ of this PEsI film (#8) was not high owing to the poor chain entanglement, as predicted from its highly linear main chains. Whereas the DK was in a common level (3.20), corresponding to an optically estimated value (*ε*_opt_ = 1.1 *n*_av_^2^ = 3.19), the combined features, a quite low DF (0.00246) and *W*_A_ (0.26%), were indeed observed.

**Figure 12:**
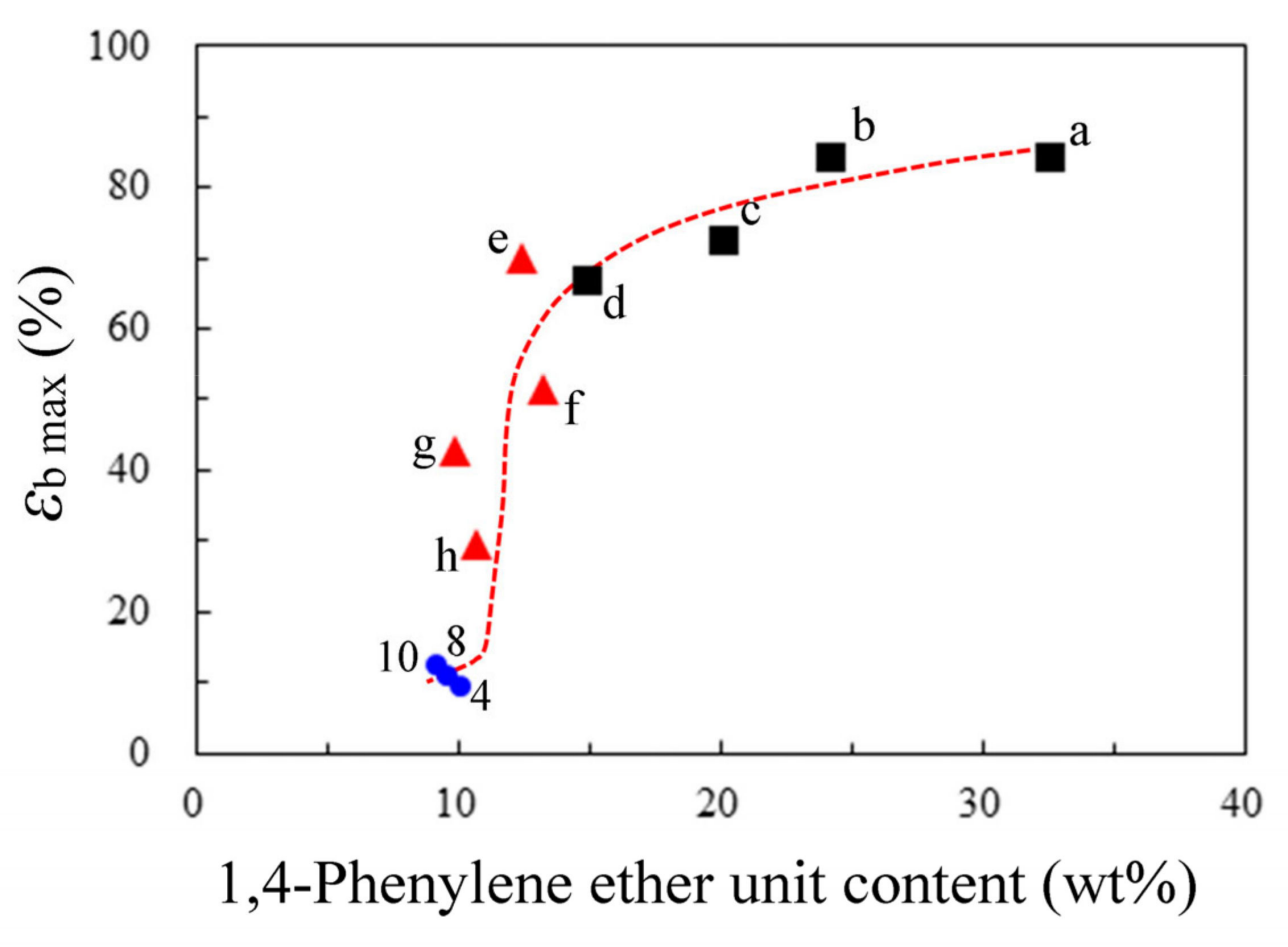
Relationship between the 1,4-phenylene ether (–Ph–O–) group content in the repeating units and the elongation at break (*ε*_b max_) for the PI and PEsI films obtained from 4,4′-ODA and various TCDAs: (a) hydroquinone diphthalic anhydride (HQDA), (b) PMDA, (c) *s*-BPDA, (d) TA-HQ, (e) TA-HPHB, (f) TA-44BP, (g) TA-44BPHB, (h) TA-HQHB, (4) TA-26NAHB, (8) TA-HQHNA, and (10) TA-26NAHNA. (■) conventional PIs, (▲) previously reported PEsIs [21], and (●) 26NA-containing PEsIs examined in this study (#4, 8, and 10).

Replacing *p*-PDA (#8) with 4,4′-ODA (#9), a significantly hazy PEsI film (haze = 62.7%) with a somewhat improved toughness (*ε*_b max_ = 12.5%) was produced. However, the observed toughening effect was not as prominent as expected, in contrast to our previous results (*ε*_b max_ = 42.9% for the counterpart derived from TA-44BPHB (*N*_Ar_ = 6) and 4,4′-ODA [21]). This likely reflects that the content of phenylene ether groups (–Ph–O–) effective for the chain entanglement was insufficient in the TA-26NAHNA/4,4′-ODA system (#9). To ascertain this assumption, the relationship between the –Ph–O– content (*C*_PhO_) and the film toughness (*ε*_b max_) was plotted for the 4,4′-ODA-based PIs and PEsIs in Figure 12. Whereas the conventional 4,4′-ODA-based PIs using common TCDAs (e.g., PMDA and *s*-BPDA) were highly tough, corresponding to their high *C*_PhO_, the film toughness tended to significantly deteriorate below *C*_PhO_ ~15 wt%. The plots of the 4,4′-ODA-based PEsIs (#4, #7, and #9) also fitted this tendency. This suggests that the unexpectedly small toughening effect observed in these systems is related to the “diluted” *C*_PhO_ by significant structural extension in these ester-linked TCDAs.

**Figure 13:**
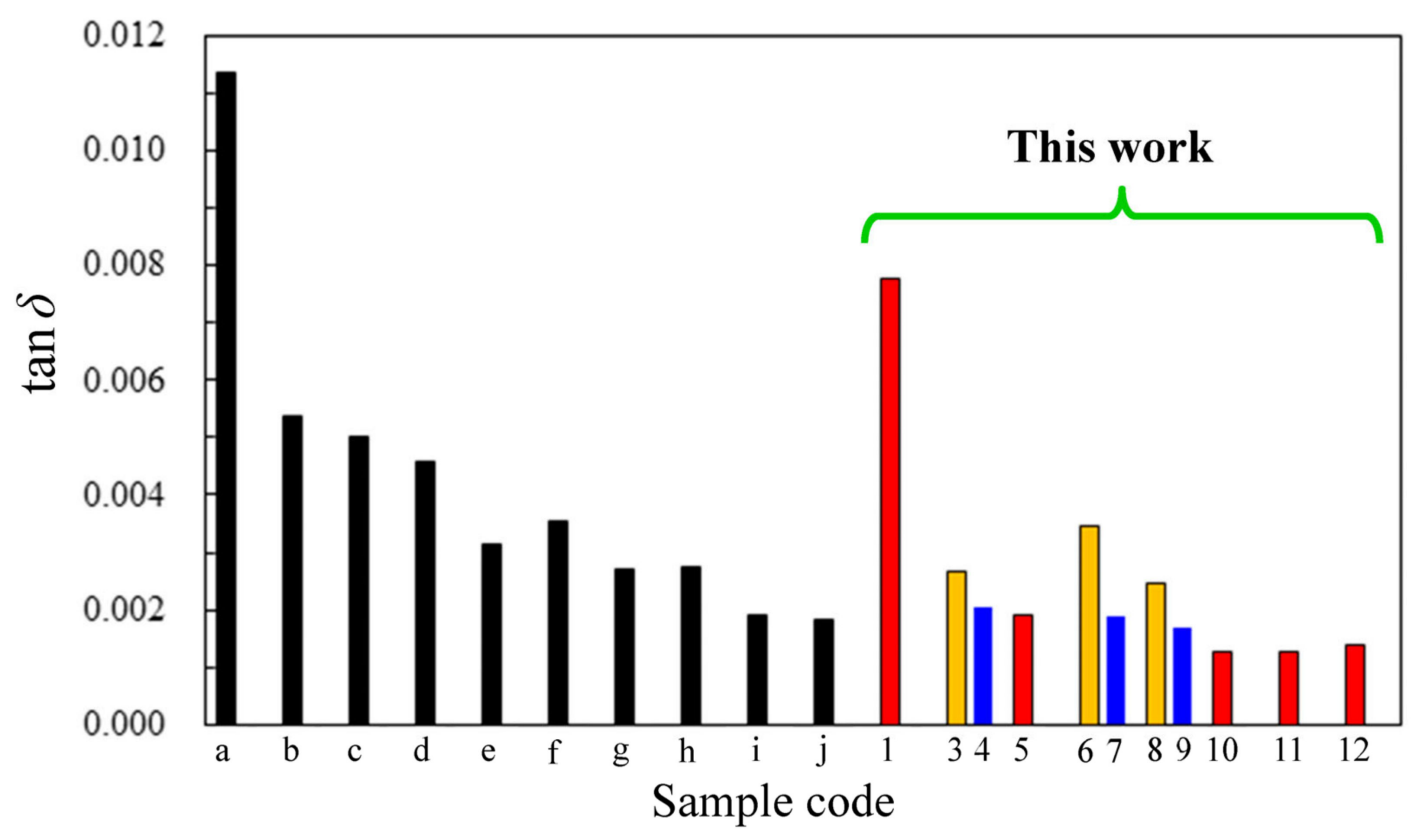
Comparison of tan *δ* (10 GHz, 50% RH, and 23 °C) for conventional PIs [black bars (a, b)], PEsIs previously reported in our studies [black bars (c–j)], and the PEsIs examined in the present study [red, orange, and blue bars (#1–12, see the sample No. in Table 4, Table 5, Table 6 and Table 7)]: (a) PMDA/4,4′-ODA, (b) *s*-BPDA/*p*-PDA, (c) TA-26NA/*p*-PDA (Table 5), (d) *s*-BPDA/APAB, (e) *s*-BPDA/MeO-ABHQ [22], (f) TA44BP/APAB, (g) TA-44BP/TFMB, (h) TA-44BPHB/*p*-PDA(75 mol%);4,4′-ODA(25 mol%) [21] (Table 5), (i) TA-HQ/BPTP (18.3 GHz, 40% RH, and 25 °C) [20], (j) TA-HNAHQ/APAB [23], (1) TA-TFMBHPBA/*p*-PDA, (3) TA-26NAHB/*p*-PDA, (4) TA-26NAHB/4,4′-ODA, (5) TA-26NAHB/*p*-PDA(75 mol%);4,4′-ODA(25 mol%), (6) TA-HQHNA/*p*-PDA, (7) TA-HQHNA/4,4′-ODA, (8) TA-26NAHNA/*p*-PDA, (9) TA-26NAHNA/4,4′-ODA, (10) TA-26NAHNA/APAB, (11) TA-26NAHNA/M-APAB, and (12) TA-26NAHNA/M-APAB (70 mol%); BAPP (30 mol%).

The use of 4,4′-ODA instead of *p*-PDA, as well as its counterparts (the related TA-26NAHB- and TA-HQHNA-based systems), also brought a positive effect on reducing DF (0.00246 → 0.00170), as listed in Table 7. The PPE-like biphenyl ether effect is shown in Figure 13 (comparison between the orange and blue bars). In addition, the 4,4′-ODA system (#9), as well as the *p*-PDA system (#8), attained both a very low *W*_A_ (0.27%) and extremely low DF (0.00170). Thus, the combined features of the TA-26NAHNA systems (Table 7, bottom) were confirmed.

#### 3.6.6. Concerns of Property Deterioration by *N*_Ar_-Increasing Approach

**Figure 14:**
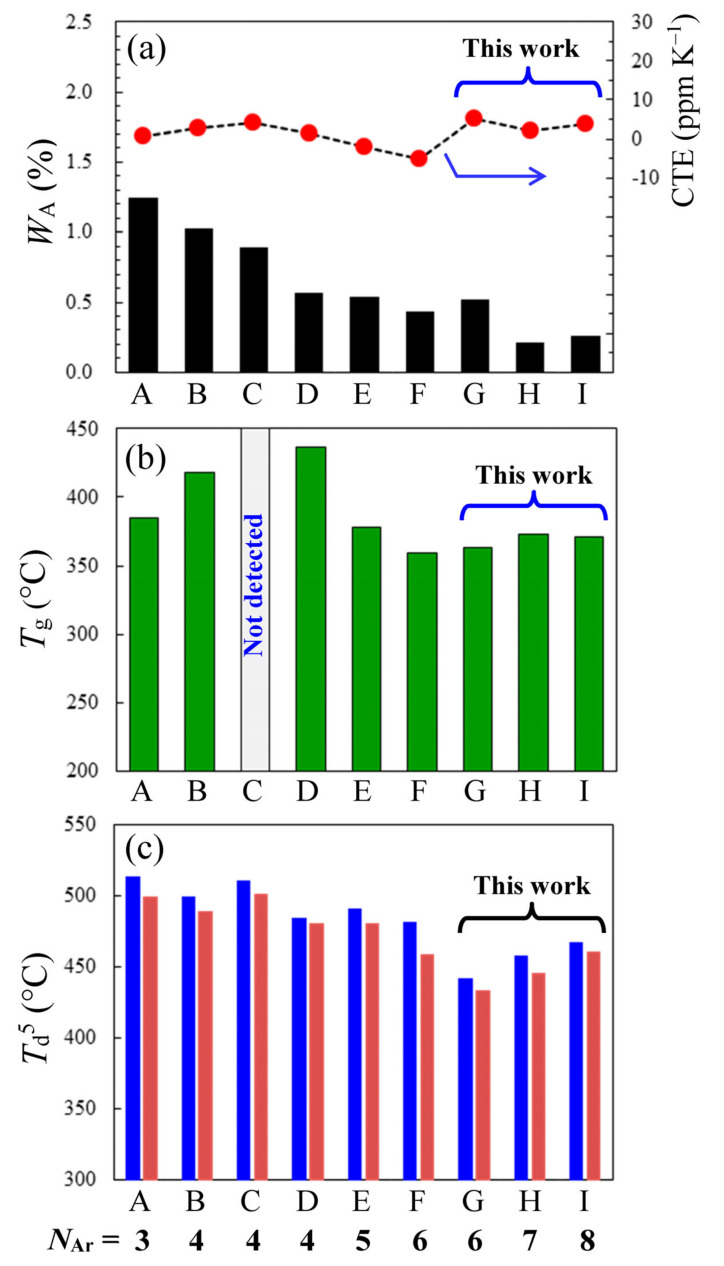
Influences of *N*_Ar_ on some specific properties: (**a**) *W*_A_ and CTE, (**b**) *T*_g_, and (**c**) *T*_d_^5^ in *N*_2_ (left, blue bars) and in air (right, red bars) for the PEsIs derived from *p*-PDA and a series of non-substituted ester-containing tetracarboxylic dianhydrides (TCDAs) with different *N*_Ar_: (A) TA-HQ, (B) TA-26NA, (C) TA-44BP, (D) TA-HPHB, (E) TA-HQHB, (F) TA-44BPHB, (G) TA-26NAHB, (H) TA-HQHNA, and (I) TA-26NAHNA. The structures and abbreviations of TCDAs are shown in Figure 8 (A and C–F), Table 5 (bottom, B), and Scheme 5, Scheme 6 and Scheme 7 (G–I).

There were other serious concerns arising from the *N*_Ar_-increasing approach: the serious deteriorations in *T*_g_, thermal stability and low CTE property [Table 3(4)–(6)]. Figure 14 shows the changes in these properties with increasing *N*_Ar_ (3–8) for the *p*-PDA-based PEsIs.

The significant in-plane chain orientation induced by thermal imidization [65,66,67,68,69], which is indispensable for the generation of a low CTE, is likely hindered by a decrease in the content of imidization-reactive sites (i.e., amic acid (AA) content) in the PAAs owing to the *N*_Ar_-increasing approach [Table 3, bottom, (a)], because imidization at the reactive sites acts as a trigger for the significant in-plane orientation. However, there was no clear trend toward a gradual increase in the CTE with increasing *N*_Ar_ [Figure 14a, broken curve]; the CTE was maintained within −5 to 5 ppm K^−1^ (specifically, CTE = 3.8 ppm K^−1^ at *N*_Ar_ = 8), whereas the *W*_A_ expectedly decreased almost monotonously with increasing *N*_Ar_ [Figure 14a, bar graph]. Thus, the concern listed in Table 3(4) was dispelled.

In addition, the *N*_Ar_-increasing approach may cause a serious decrease in *T*_g_ owing to a concomitantly decreased *C*_i_ [Table 3, bottom, (b)], because the dipole–dipole interchain interaction between the imide C=O groups [75,76] is believed to be the origin of the strong intermolecular forces in PIs. However, no clear tendency toward a gradual decrease in *T*_g_ [Table 3(5)] was observed, as shown in Figure 14b (specifically, *T*_g_ = 371 °C even for *N*_Ar_ = 8).

Another concern with increasing *N*_Ar_ is a serious decrease in the thermal stability (*T*_d_^5^ in N_2_) [Table 3(6)], which can occur with a concomitant increase in the content of the ester group, which is thermally less stable than the commonly used aromatic ether connecting groups. Indeed, a gradual decrease in *T*_d_^5^ (in both N_2_ and air) was observed [Figure 14c]. However, even the PEsI with *N*_Ar_ = 8 maintained a sufficiently high *T*_d_^5^ value in N_2_ (467 °C).

Thus, there were virtually no serious concerns regarding the *N*_Ar_-increasing approach for *N*_Ar_ = 6–8.

#### 3.6.7. Attempt to Further Reduce DF

We attempted to further reduce the DF of the TA-26NAHNA-based PEsIs using simple ester-linked diamines (APAB and M-APAB). The thermally imidized films were ductile with resistance to the zero-*r*_c_ bending tests. The properties of these PEsI films are listed in Table 7. The APAB system (#10) produced a hazy PEsI film (haze = 42.2%), as shown at the bottom (left) of Table 7. This PEsI film exhibited a very high *T*_g_ (358 °C) and a very low CTE (11.3 ppm), corresponding to a high Δ*n*_th_ (0.110), and a very low *W*_A_ (0.24%) as in the other TA-26NAHNA-based counterparts. Although the DK (3.05) was somewhat lower than the common level, an ultra-low DF (0.00128), which was lower than the temporal goal (DF = 0.002 for LCPs), was obtained. This PEsI film (#10) also maintained sufficiently high thermal stability [*T*_d_^5^ (N_2_) = 450 °C].

The use of methyl-substituted APAB (M-APAB, #11) afforded an almost clear PEsI film (haze = 12.2%), as shown at the bottom (center) of Table 7, with excellent combined properties except for the film toughness: an exceedingly high *T*_g_ (365 °C) with a weak *β*-transition (178 °C), extremely low CTE (6.8 ppm K^−1^), and ultra-low DF (0.00128).

To improve the film toughness of the M-APAB system (#11), 2,2-bis[4-(4-aminophenoxy)phenyl]propane (BAPP), which is often more effective in toughening PI films than 4,4′-ODA was copolymerized with a minor content (30 mol%) to avoid significant deteriorations of the CTE and *T*_g_. The resulting copolymer (#12) afforded a visually completely clear PEsI film (haze = 3.8%), as shown at the bottom (right) of Table 7, with a slightly improved toughness (*ε*_b max_ = 7.2%), although its toughening effect was not as pronounced as expected. Despite the partial use of flexible BAPP, the copolymer retained a very high *T*_g_ (349 °C) and a low CTE (16.0 ppm K^−1^) close to that of copper foils. This PEsI film also achieved excellent combined properties of an extremely low *W*_A_ (0.22%), relatively low DK (2.85), and ultra-low DF (0.00138).

Thus, the combinations of TA-26NAHNA and simple ester-linked diamines (APAB and M-APAB) afforded promising candidates for unprecedented 5G-compatible dielectric film materials that achieved exceedingly high *T*_g_s, extremely low CTEs, and ultra-low DFs simultaneously.

#### 3.6.8. Structure–DF Relationship

Figure 13 compares the DF values (10 GHz, 50% RH, and 23 °C) of the PEsIs and related systems for examining the structure–DF relationship. It is clear from this figure that the present PEsIs (particularly, #10, #11, and #12) had ultra-low DF.

The PEsI (c), derived from *p*-PDA and a simple ester-containing TCDA (TA-26NA, Table 5, bottom, *N*_Ar_ = 4), had a still high DF (0.00501), although it was lower than those of PMDA/4,4′-ODA (a) (DF = 0.0114) and *s*-BPDA/*p*-PDA (b) (DF = 0.00538). Thus, the DF-reducing effect of TA-26NA was limited, probably because of its insufficient structural extension. Comparing *s*-BPDA/APAB (d) with *s*-BPDA/methoxyhydroquinone bis(4-aminobenzoate) (MeO-ABHQ) (e) [22], the latter exhibited a clearly decreased DF owing to the *N*_Ar_-increasing effect of the diamine [MeO-ABHQ (*N*_Ar_ = 3)] used in the latter. Comparing *s*-BPDA/APAB (d) with TA-44BP/APAB (f), the decreased DF of the latter is undoubtedly attributed to the increased *N*_Ar_ of TA-44BP.

TA-44BP/TFMB (g) showed a clearly decreased DF compared to TA-44BP/APAB (f), reflecting the extremely low polarizability of the CF_3_ groups in the former. The combinations of ester-containing TCDAs and ester-containing diamines, that is, TA-HQ/BPTP(i) [20] and TA-HNAHQ/APAB (j) [23], were significantly effective in reducing the DF.

Comparing the DF values of the APAB-based related systems, it is evident how effective the TA-26NAHNA developed in this study is for dramatically reducing DF in the following order: *s*-BPDA/APAB (d) > TA-44BP/APAB (f) >>TA-26NAHNA/APAB (#10).

### 3.7. Factors Dominating DF and W_A_

#### 3.7.1. Impact of Imide Group Content

**Figure 15:**
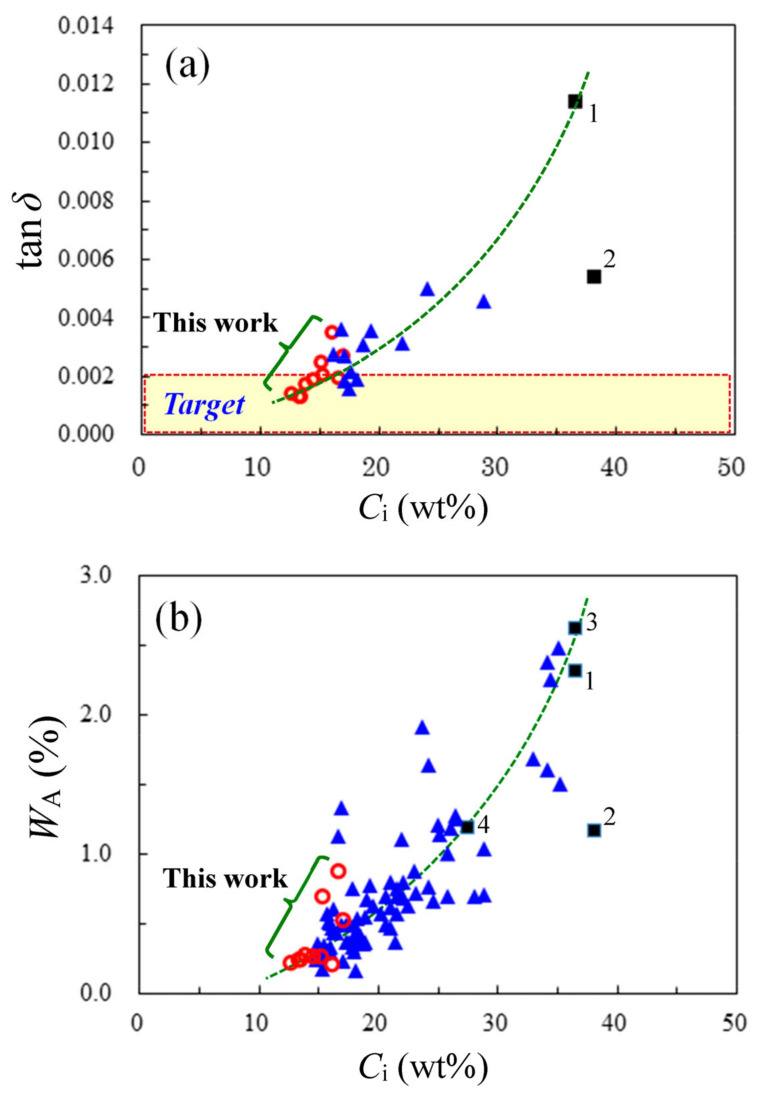
Correlation of the imide group content (*C*_i_) with tan *δ* (10 GHz, 50% RH, and 23 °C) (**a**) and water uptake (*W*_A_) (**b**) for PI and PEsI films: (■) conventional wholly aromatic PIs [1: PMDA/4,4′-ODA, 2: *s*-BPDA/*p*-PDA, 3: PMDA/3,4′-ODA), and 4: PMDA/4,4′-ODA(40);BAPP(60)], (▲) PEsIs examined in our previous studies, and (**○**) PEsIs developed in this work. The data for fluorine- and amide-containing systems are excluded from this figure.

Figure 15a shows the relationship between DF and *C*_i_ for the PEsIs and conventional PIs. A correlation, in which DF monotonously decreases with decreasing *C*_i_, was observed with a somewhat scattering of the plots. A part of the plots for the 26NA-containing PEsIs developed in this study (red open circle, ○) were located to the lower left of those of our previously reported PEsIs (blue solid triangle, ▲). Thus, thoroughly reducing the highly polarized imide groups in the structures was the direct approach to actualize these ultra-low DFs. A temporal goal (DF = 0.002 for LCPs) was achieved below *C*_i_~15 wt% in the PEsIs. Figure 15a also suggests that the ester connecting groups, in contrast to the imide groups, did not significantly involve an increase in DF, because a decrease in *C*_i_ corresponds to an increase in the content of the ester connecting groups.

In this figure, the plot of *s*-BPDA/*p*-PDA (■, No.2) significantly deviates downward from the guiding curve representing the correlation. This likely reflects a more limited free volume in *s*-BPDA/*p*-PDA than in the other systems, corresponding to the fact that, when this PI film was prepared via heat treatment above 400 °C, partial crystallization occurred with a significantly increased density [66].

Figure 15b shows the relationship between *W*_A_ and *C*_i_. A correlation, in which *W*_A_ monotonously reduces with decreasing *C*_i_, was observed in the somewhat scattered plots. As similarly in Figure 15a, a part of the plots for the 26NA-containing PEsIs developed in this study (red open circle, **○**) were located to the lower left of those of our previously reported PEsIs (blue solid triangle, ▲), suggesting that thoroughly lowering *C*_i_ is the primary strategy for achieving extremely low *W*_A_. Similar to the above-mentioned DF–*C*_i_ relationship, the ester connecting group did not significantly participate in the water-absorbing behavior of the PEsI films. A similar significant downward deviation of the plot of the *s*-BPDA/*p*-PDA system (■, No.2) can also be explained by its limited free volume.

#### 3.7.2. Correlation between DF and W_A_

**Figure 16:**
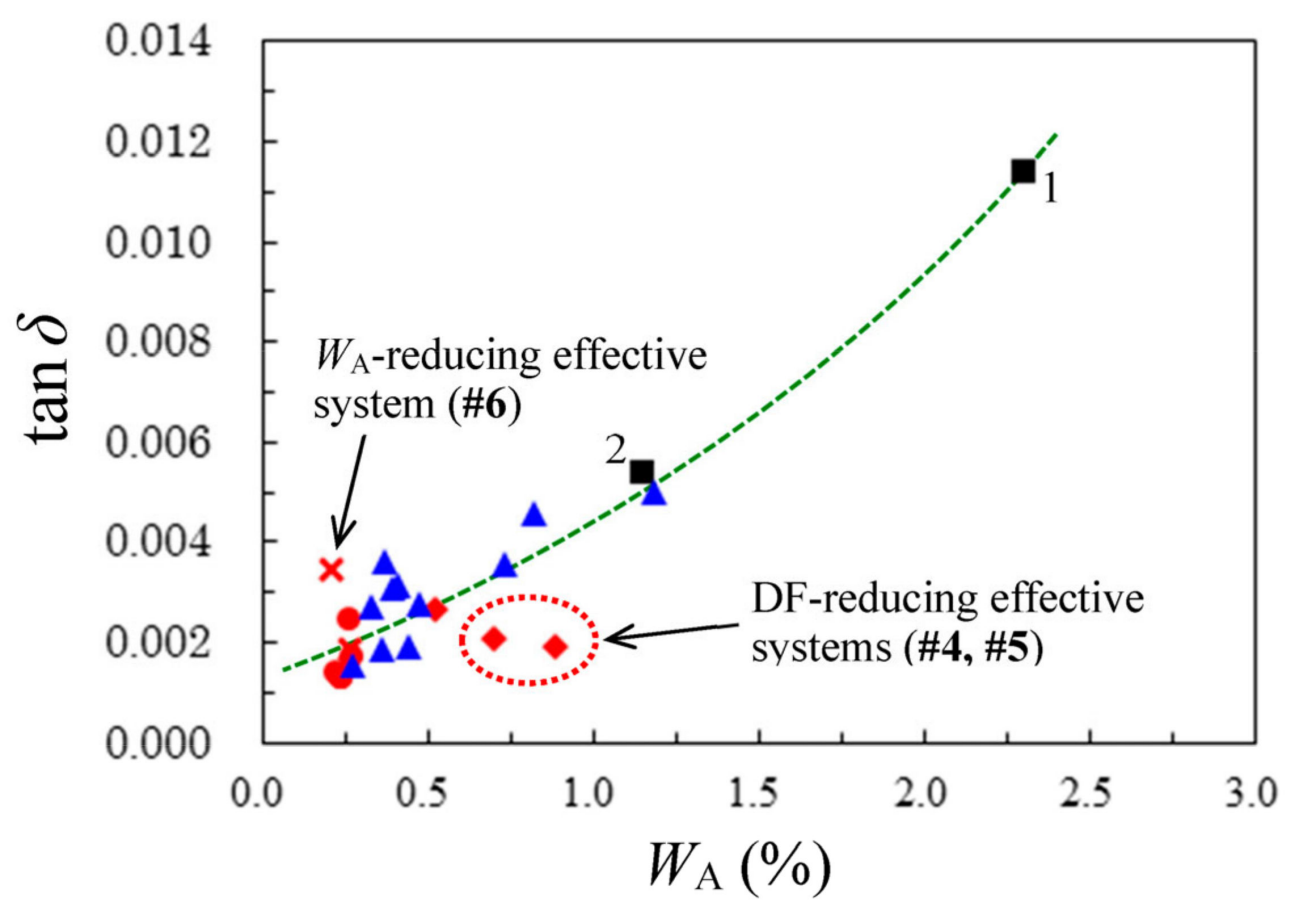
Relationship between water uptake (*W*_A_) and tan *δ* (10 GHz, 50% RH, and 23 °C) for conventional PI and PEsI films: (■) conventional wholly aromatic PIs (1: PMDA/4,4′-ODA, 2: *s*-BPDA/*p*-PDA), (▲) PEsIs examined in our previous studies, and (red symbols, ♦, ×, **●**) PEsIs developed in this work: (♦) TA-26NAHB-based PEsIs, (×) TA-HQHNA-based PEsI, and (**●**) TA-26NAHNA-based PEsIs. The data for fluorine- and amide-containing systems are excluded from this figure.

Figure 16 shows the relationship between DF and *W*_A_ for the PEsIs and conventional PIs. A better correlation than in Figure 15 was observed. In particular, the significant downward deviations of the *s*-BPDA/*p*-PDA plot (■, No.2) in the DF–*C*_i_ and *W*_A_–*C*_i_ relationships were virtually canceled. This suggests that *W*_A_ is a better index for DF over a wide range of materials from PIs to PEsIs. Figure 16 also clearly shows the distinct downward deviations of the plots (♦) for the TA-26NAHB-based systems (#4, #5), which confirm again the above-mentioned DF-reducing effect of TA-26NAHB. On the other hand, the *W*_A_-reducing effect of the TA-HQHNA-based system (#6) is evident from the significant leftward deviation of the plot (×).

#### 3.7.3. Review of DF- and *W*_A_-Controlling Factors

**Figure 17:**
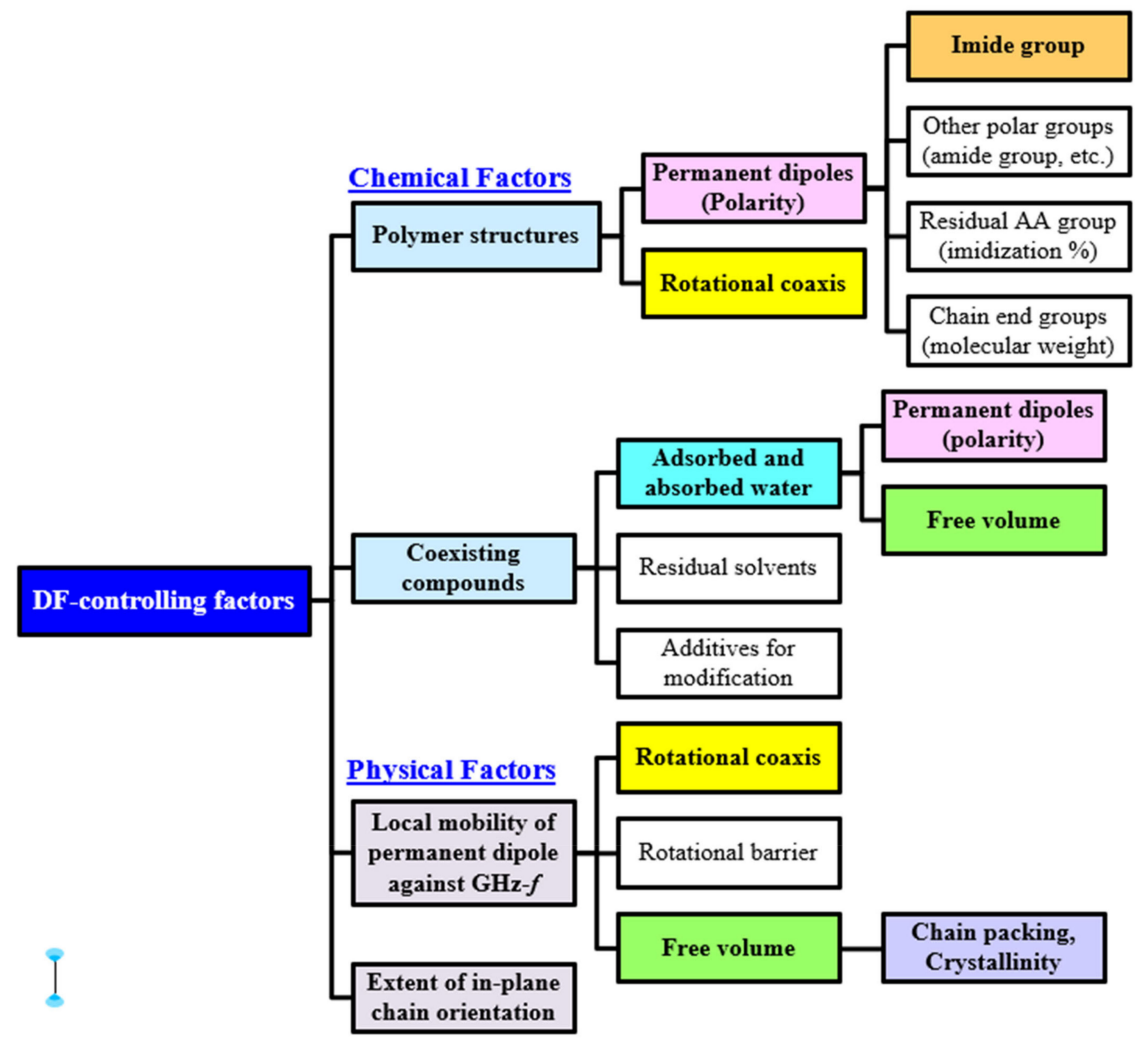
Key factors influencing the DF values of the PI and PEsI films.

The good correlation observed between DF and *W*_A_ in Figure 16 suggests that the DF-controlling factors are virtually the same as or very similar to the *W*_A_-controlling factors. To ascertain this assumption, these factors were comprehensively reviewed according to Figure 17.

There are chemical and physical factors for controlling DF. The former is classified as originating from polymer structures and low-molecular-weight compounds contained in the polymer films. The factors related to the polymer structures include the strength and content of permanent dipoles in the polymer structures and the content of the rotational coaxis. The parameters associated with permanent dipoles consist of *C*_i_ and, if any, the contents of polar groups other than imide groups (e.g., amide group), residual unimidized (AA) portions, and chain ends (in the case of insufficient molecular weights). These factors (except for *C*_i_), which come from defective specimens, are excluded in this study (these are not highlighted in color in Figure 17).

The coexisting compounds (particularly, adsorbed/absorbed water) also significantly influence the DF. Other possible coexisting compounds are residual solvents and various additives, which were excluded from this study. The *W*_A_ is undoubtedly closely related to the polarity of the polymer structure and the water-entering free volume.

The primary physical factor is the local and small rotational motion (wagging motion) in response to high-*f* electric fields. The in-plane chain orientation is also included in the physical factors, although it was not discussed in this study. The wagging motion is affected by the content of the rotational coaxis, rotational barriers (size of the wagging units, intramolecular steric hindrance, and intermolecular forces), and free volume around the wagging units, which is indispensable for wagging motion.

Figure 17 also indicates that the *W*_A_-controlling factor (highlighted in light blue) is associated with polymer polarity (highlighted in pink) and free volume (highlighted in green). Therefore, *W*_A_ almost covers the primary chemical and physical factors for DF control, as shown in this figure. This is a reasonable reason for the good correlation observed between the DF and *W*_A_ in Figure 16.

### 3.8. Performance Balance of Our PEsIs

Practically, it is essential to achieve the required properties in a well-balanced manner. However, it has thus far been very difficult to simultaneously achieve exceedingly high *T*_g_, extremely low CTE, and ultra-low DF in addition to other required properties, as illustrated by the fact that existing ultra-low-DF materials (PTFE, PE, COP, etc.) do not have high *T*_g_ and low CTE.

The performance balance of certain typical PEsIs developed in this study was overviewed using their spider charts, which were prepared based on the five-step-ranked achievement level of each required item (Table 8). For example, the samples with *T*_g_ ≥ 360 °C (or non-detectable *T*_g_ by DMA) were ranked 5 (highest) with reference to *T*_g_ = 360–370 °C for *s*-BPDA/*p*-PDA with virtually the highest *T*_g_ among common PIs [77]. On the other hand, *T*_g_ ≤ 210 °C was ranked 1 (lowest) with reference to *T*_g_ = 215 °C for poly(ether imide) derived from bisphenol A-type TCDA and *m*-PDA, which has virtually the lowest *T*_g_ among common PIs [78]. The span (360–210 °C), as well as other required items, was evenly allocated to an intermediate rank (4–2).

CTE ≤ 10 ppm K^−1^ was ranked 5 with reference to CTE = 5–15 ppm K^−1^ for *s*-BPDA/*p*-PDA with virtually the lowest CTE among common PIs [65,66], and CTE ≥ 70 ppm K^−1^ was ranked 1 with reference to the CTEs (50–70 ppm K^−1^) for many flexible PIs and common polymers [31].

*ε*_b max_ ≥ 80% was ranked 5 with reference to *ε*_b_~80% for PMDA/4,4′-ODA with virtually the highest *ε*_b_ among common PIs [30], and *ε*_b max_ ≤ 2% as in the PMDA/*p*-PDA system or none of the film-forming ability (generation of cracks) was ranked 1.

*W*_A_ ≤ 0.1% was ranked 5 with reference to *W*_A_ = 0.2% for a typical fluorinated PI derived from 4,4′-(hexafluoroisopropylidene)diphthalic anhydride (6FDA) and TFMB [35], and *W*_A_ ≥ 3.0% was ranked 1 with reference to *W*_A_ = 3.4% for PMDA/DABA with virtually the highest *W*_A_ among common PIs.

Haze ≤ 2% with a visually complete clearness was ranked 5, and haze ≥ 50%, under which it becomes difficult to visually recognize printed characters through the films, was ranked 1.

Regarding dielectric properties measured at 10–20 GHz, *ε*_r_ ≤ 2.7 was ranked 5, and *ε*_r_ ≥ 3.7 was ranked 1. On the other hand, tan *δ* ≤ 0.001 was ranked 5, and tan *δ* ≥ 0.02 was ranked 1. These were ranked based on the fact that many common polymers have an *ε*_r_ ranging from 2.01 to 5.0, and tan *δ* ranging from 0.00023 to 0.02 [31].

Specifically, each required item was ranked as shown in Table 8. However, the achievement level for each required item tends to become more severe every year. Concomitantly, these rankings will be gradually updated.

**Figure 18:**
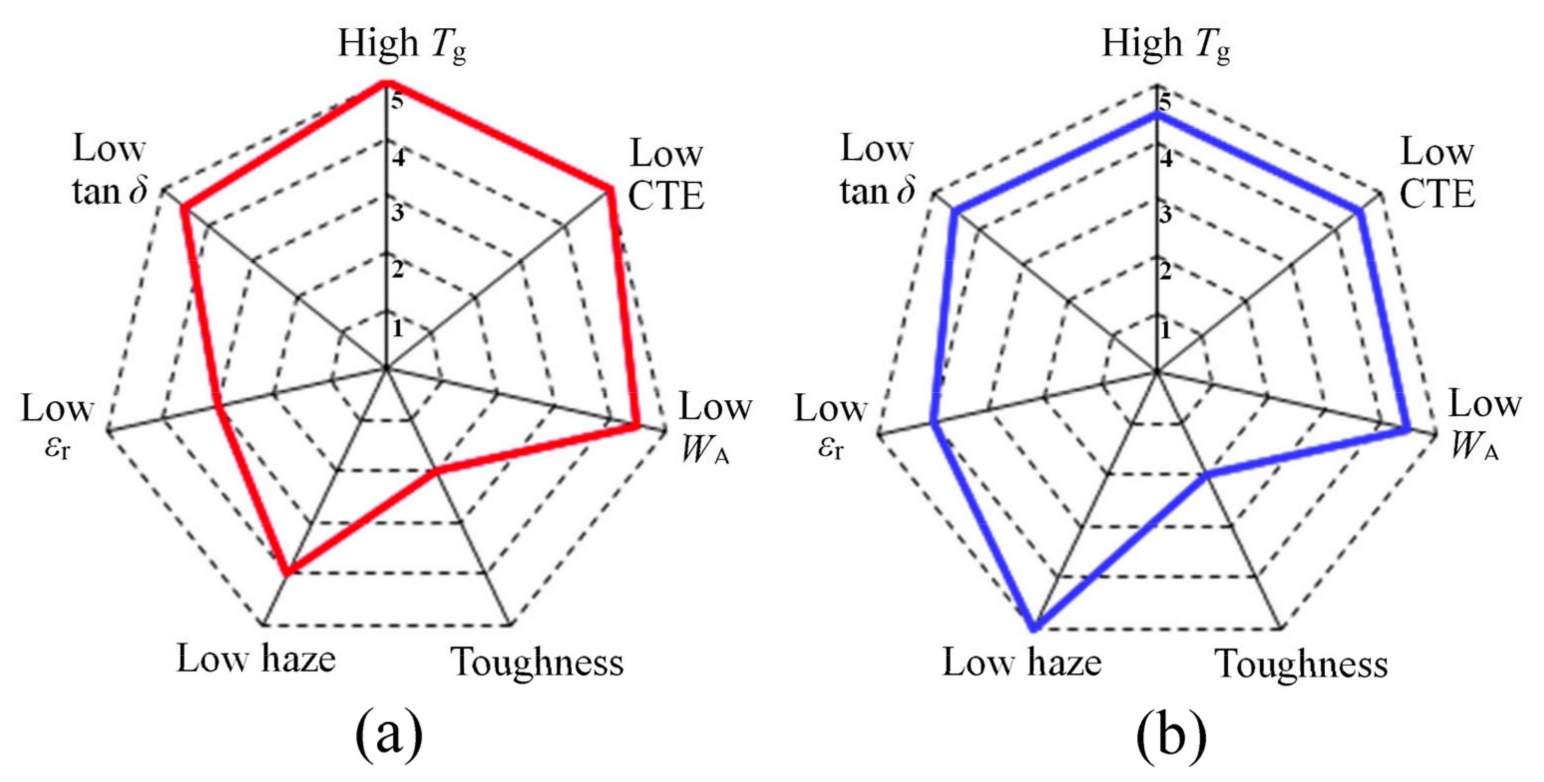
Performance balance based on five-rank criteria (Table 8) for the PEsIs examined in this study: (**a**) TA-26NAHNA/M-APAB and (**b**) TA-26NAHNA/M-APAB(70);BAPP(30) systems.

For example, Figure 18a shows a spider chart for the TA-26NAHNA/M-APAB system (#11). This PEsI film achieved excellent combined properties: an exceedingly high *T*_g_, extremely low CTE, very low *W*_A_, and low haze, although there was room for improvement in its DK and film toughness (particularly, the latter).

On the other hand, the copolymer system (#12), which was obtained by modifying the TA-26NAHNA/M-APAB system (#11) via copolymerization using BAPP (30 mol%), exhibited a further improved *ε*_r_ and haze and a slightly improved *ε*_b_ while maintaining the original excellent combined properties of the pristine system (#11). Thus, the copolymerization approach improved the performance balance, as shown in Figure 18b, although the toughness should be further improved. However, these PEsI films were not hopelessly brittle with resistance to the zero-*r*_c_ bending tests. In addition, it is worth noting that these PEsI films were produced using the conventional two-step process without any complicated stretching processes, such as the inflation method, which is often used in LCP systems. Therefore, these PEsIs are promising candidates as dielectric films for use in 5G-compatible high-performance FPCs.

## 4. Conclusions

This study attempted to develop new dielectric films achieving an exceedingly high *T*_g_, very low CTE, ultra-low DF, and other required properties simultaneously, aiming to apply them to 5G-compatible high-performance FPCs. It was suggested that the only limited solution is to obtain PEsIs using TCDAs with longitudinally *para*-linked extended structures via ester connecting groups. However, the synthesis of an initially desired TCDA with *N*_Ar_ = 8 (TA-44BPHPBA), including multiple 4,4′-biphenylene units, was unsuccessful owing to precipitation of a mono-acetoxide intermediate based on its complete insolubility.

Strategies to avoid this issue were proposed: (1) introduction of two CF_3_ groups to the central 4,4′-biphenylene unit in the TCDA using TFMB as the raw material and (2) replacement of the 4,4′-biphenylene units with the slightly bulkier 26NA units. These approaches effectively improved the solubility of the intermediates and enabled us to synthesize a series of novel *para*-ester-linked TCDAs with longitudinally extended structures: (a) TA-TFMBHPBA (*N*_Ar_ = 8), (b) TA-26NAHB (*N*_Ar_ = 6), (c) TA-HQHNA (*N*_Ar_ = 7), and (d) TA-26NAHNA (*N*_Ar_ = 8).

These TCDAs showed essentially high polyaddition reactivity with common diamines such as *p*-PDA and 4,4′-ODA and led to homogeneous solutions of PAAs with sufficiently high *η*_red_ values (molecular weights). Thermal imidization of the resulting PAA cast films essentially afforded ductile PEsI films, except for a part of these systems, as suggested by the resistance to the zero-*r*_c_ bending tests. These PEIs have the following features.

(a) TA-TFMBHPBA-based PEsI systems

As an example, the TA-TFMBHPBA/*p*-PDA system (#1) exhibited an exceedingly high *T*_g_ (395 °C), high tensile modulus (5.76 GPa), and a relatively low CTE (27.5 ppm K^−1^), reflecting its stiff/linear chain structure. In addition, this PEsI (#1), despite the presence of highly polarized amide groups in its main chain, retained a relatively low *W*_A_ (0.70%). This likely reflects that the high polarity of the amide groups was “diluted” by the adjacent less polar CF_3_ groups. In contrast, the DF remained relatively high (0.00776). This suggests that the negative impact of the amide groups on the DF was not easily canceled out by the adjacent CF_3_ groups.

(b) TA-26NAHB-based PEsI systems

As an example, the TA-26NAHB/*p*-PDA system (#3) exhibited a very high *T*_g_ (366 °C), ultra-low CTE, reflecting its stiff/linear main chains, in addition to a relatively low *W*_A_ (0.52%). Whereas its DK was at a common level (3.22), the DF was an extremely low value (0.00267). Thus, the TA-26NAHB systems had a feature: the DF-reducing effect was more pronounced than the *W*_A_-reducing effect.

The TA-26NAHB/*p*-PDA(75);4,4′-ODA(25) copolymer exhibited a clearly decreased DF (0.00191), compared with that of its counterpart where the central 26NA unit of the former was replaced with the 4,4′-biphenylene unit. The result was difficult to explain by the difference in their *C*_i_ values because the former has a slightly higher *C*_i_ than the latter. An alternative possible explanation was proposed: the wagging motions of the central 26NA units are restricted compared to those of the 4,4′-biphenylene unit, owing to the absence of the rotational coaxis and inter-stack between the planar 26NA units in the former.

(c) TA-HQHNA-based PEsI systems

For example, the TA-HQHNA/*p*-PDA system (#6) has a very high *T*_g_ (373 °C) and ultra-low CTE (1.9 ppm K^−1^). Although the DF was not significantly reduced, compared to that of its counterpart (#3), a dramatic reduction in the *W*_A_ (0.21%) was observed. Thus, the TA-HQHNA system had a feature: the *W*_A_-reducing effect was much more pronounced than the DF-reducing effect.

(d) TA-26NAHNA-based PEsI systems

For example, TA-26NAHNA/*p*-PDA (#8) achieved multiple excellent properties: a considerably high *T*_g_ (371 °C), ultra-low CTE (3.8 ppm K^−1^), and very high modulus (7.70 GPa). This system also simultaneously achieved an extremely low DF (0.00246) and a very low *W*_A_ (0.26%), which is the combination of each feature in the TA-26NAHB and TA-HQHNA systems. The use of 4,4′-ODA (#9) somewhat improved the film toughness; however, its effect was not as pronounced as expected, as observed similarly in the other systems (TA-TFMBHPBA, TA-26NAHB, and TA-HQHNA systems). This is probably caused by an extremely decreased phenylene ether content in the repeating unit of TA-26NAHB/4,4′-ODA, which originates from the significantly increased TCDA molecular weight by the *N*_Ar_-increasing approach.

The present PEsI systems (*N*_Ar_ = 6–8 in the TCDIs) were compared with our previously reported PEsIs (*N*_Ar_ ≤ 6). The *N*_Ar_-increasing approach caused no clear tendency toward the gradual deterioration of CTE and *T*_g_, in contrast to a gradual decrease in *W*_A_. However, a gradual decrease in the thermal stability [*T*_d_^5^ (N_2_)] with increasing *N*_Ar_ was unavoidably observed, owing to a concomitant increase in the content of the ester connecting group with its relatively low bond energy. However, even the TA-26NAHNA-based PEsIs (*N*_Ar_ = 8) maintained a sufficiently high *T*_d_^5^ (N_2_). Thus, our initial concerns, that is, serious property deterioration caused by the *N*_Ar_-increasing approach, were virtually dispelled.

This study also discussed the DF-controlling factors. A DF–*C*_i_ correlation, in which DF monotonously decreases with increasing *C*_i_, was observed with a somewhat scattering of the plots. This also suggests that the ester connecting groups do not significantly involve the increase in DF. The scattering of these plots probably suggests that the physical factors, such as the wagging motions of permanent dipoles and the free volume around them, are not reflected in this relationship. A similar correlation was observed between *W*_A_ and *C*_i_ with somewhat scattering in the plots. The physical factors such as the free volume around the imide groups are also not reflected in this relationship. In contrast, a better correlation was observed between DF and *W*_A_. This suggests that *W*_A_ is a better index for DF than *C*_i_ over a wide range of materials from PIs to PEsIs. To explain the observed better correlation between the DF and *W*_A_, the commonality of the DF- and *W*_A_-controlling factors was discussed in this study.

(e) PEsIs derived from TA-26NAHNA and ester-linked diamines

For example, the TA-26NAHNA/M-APAB system (#11) afforded a ductile and almost clear PEsI film (haze = 12.2%). This PEsI achieved excellent combined properties: a considerably high *T*_g_ (365 °C), ultra-low CTE (6.8 ppm K^−1^), very low *W*_A_ (0.24%), and ultra-low DF (0.00128). However, there was room for improvement in the film toughness. BAPP was then copolymerized with #11 in a minor content (30 mol%). The resulting copolymer (#12) produced a ductile and visually completely clear PEsI film (haze = 3.8%). A certain toughening effect of BAPP was observed (*ε*_b max_ = 7.2%), although its effect was not as pronounced as expected. Despite the use of flexible BAPP, the copolymer maintained excellent combined properties: a very high *T*_g_ (349 °C), low CTE (16.0 ppm K^−1^) close to that of copper foils, very low *W*_A_ (0.22%), relatively low DK (2.85), and ultra-low DF (0.00138).

Thus, certain PEsI films developed in this study are promising candidates as dielectric films for use in 5G-compatible high-performance FPCs.

## Figures and Tables

**Table 1:** Abbreviations, commercial sources, vacuum-drying conditions before use, and melting points of the common monomers used in this study.

Monomer	Source	Vacuum-Drying Condition	Melting Point ^a^ (°C)
*p*-Phenylenediamine (*p*-PDA)	Wako Chemical	30 °C/12 h	141
4-Aminophenyl 4-aminobenzoate (APAB)	Wakayama Seika	50 °C/12 h	185
4-Amino-2-methylphenyl 4-aminobenzoate (M-APAB)	Wakayama Seika	50 °C/12 h	162
4,4′-Oxydianiline (4,4′-ODA)	Wako Chemical	50 °C/12 h	191
2,2-Bis[4-(4-aminophenoxy)phenyl]propane (BAPP)	Wakayama Seika	50 °C/12 h	130

^a^ Data determined from the endothermic peak temperature measured at a heating rate of 5 °C min^−1^ on DSC.

**Table 2:** Abbreviations, commercial sources, and melting points of the raw materials used in this study.

Raw Material	Source	Melting Point (°C)
Hydroquinone (HQ)	Wako Chemical	173 ^a^
4,4′-Biphenol (44BP)	Tokyo Chemical Industry (TCI)	286 ^a^
2,6-Dihydroxynaphthalene (26DHNA)	TCI	222 ^b^
2,2′-Bis(trifluoromethyl)benzidine (TFMB)	Wakayama Seika	184 ^a^
4-(4-Hydroxyphenyl)benzoic acid (HPBA)	Sanko (or TCI)	294 ^c^
6-Hydroxy-2-naphthoic acid (HNA)	TCI	248 ^b^
4-Hydroxybenzoic acid (HBA)	TCI	215 ^b^
4-Acetoxybenzoic acid (AcBA)	TCI	193 ^b^
Trimellitic anhydride chloride (TMAC)	TCI	69 ^a^

^a^ Data determined from the endothermic peak temperature measured at a heating rate of 5 °C min^−1^ on DSC. ^b^ Data from the safety data sheet. ^c^ Sanko Co., Ltd., URL: https://sanko-chem.com/own-products/ppp-cooh-2/ (accessed on 19 February 2024).

**Table 3:** Predicted problems arising from longitudinal structural extension of all-*para*-linked ester-containing tetracarboxylic dianhydrides (TCDAs). The schematic drawings at the bottom of this table show the decreased contents of the solvating groups (=imidization reactive groups) in the PAAs (a) and the imide groups (b) in the PEsIs by structural extension of ester-linked aromatic units (Ar).

	Possible Obstacles	Specific Concerns	Possible Countermeasures
(1)	Monomer synthesis	Complete insolubility of intermediates of TCDAs	Introduction of adequate substituents, more bulky or asymmetric aromatic units
(2)	Polyaddition	No reaction due to complete insolubility of TCDAs	Dilution or softheating during polyaddition
(3)	ibid	Gelation or precipitation of PAA solutions during polyaddition due to a decrease in the COOH/NHCO groups	Dilution or softheating after polyaddition
(4)	ibid	CTE ↑ due to a decrease in the AA content for imidization	None
(5)	Deterioration of PEsI properties	*T*_g_ ↓ due to a decrease in the imide group content	None
(6)	ibid	*T*_d_ ↓ due to an increase in the ester group content	None

↑ and ↓ represent an increase and decrease in each category. 
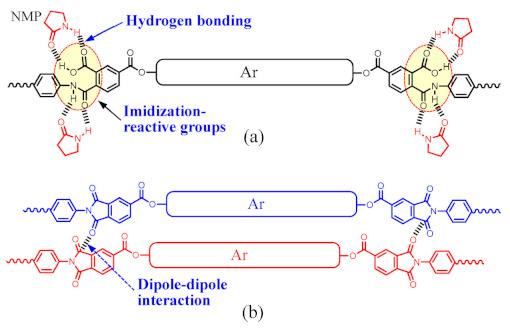

**Table 4:** Film properties of PEsIs derived from TA-TFMBHPBA (*N*_Ar_ = 8) with common aromatic diamines. The structure of TA-TFMBHPBA is shown at the bottom of this table.

No.	Diamine	*η*_inh_ PAA (dL g^−1^)	*T*_g_ (°C)	CTE (ppm K^−1^)	*E* (GPa)	*ε*_b_ av/max (%)	*σ*_b_ (GPa)	*T*_d_^5^ in N_2_ (°C)	*T*_d_^5^ in Air (°C)	*W*_A_ (%)	*ε*_r_ @10 GHz	tan *δ* @10 GHz
1	*p*-PDA	2.42	395 ^a^ (200) ^c^	27.5	5.76	2.8 4.4	0.11	476	456	0.70	3.34	0.00776
2	4,4′-ODA	2.47	377 ^a^ 393 ^b^ (199) ^c^	33.4	3.43	3.7 7.7	0.06	484	456	1.03	---	---

^a^ *T*_g_ determined by DMA. ^b^ *T*_g_ determined by TMA. ^c^ *T*_β_ determined by DMA. 
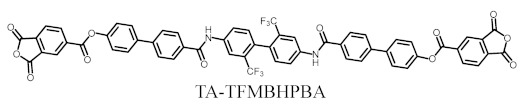

**Table 5:** Film properties of PEsIs derived from TA-26NAHB (*N*_Ar_ = 6) and common aromatic diamines and related systems. The structures of TA-26NAHB and its analogs (TA-44BPHB and TA-26NA) are shown at the bottom of this table.

No.	Diamine (mol%)	*η*_inh_ PAA (dL g^−1^)	*T*_g_ (°C)	CTE (ppm K^−1^)	*E* (GPa)	*ε*_b_ av/max (%)	*σ*_b_ (GPa)	*T*_d_^5^ in N_2_ (°C)	*T*_d_^5^ in air (°C)	*W*_A_ (%)	*ε*_r_ @10 GHz	tan *δ* @10 GHz
3	*p*-PDA	2.16	363 ^a^ 395 ^b^	5.1	--- ^d^	--- ^d^	--- ^d^	442	433	0.52 (1.03) ^f^	3.22 (3.22) ^f^	0.00267 (0.00501) ^f^
4	4,4′-ODA	1.45	250 ^b^ (163) ^c^	55.0 (23.7) ^e^	2.55	6.5 9.6	0.07	442	433	0.70	3.01	0.00206
5	*p*-PDA (75) 4,4′-ODA(25)	1.33	362 ^a^ 377 ^b^ (174) ^c^	10.5	4.83	5.6 11.9	0.14	445	433	0.88 (0.47) ^g^	3.06 (3.22) ^g^	0.00191 (0.00276) ^g^

^a^ *T*_g_ determined by DMA. ^b^ *T*_g_ determined by TMA. ^c^ *T*_β_ determined by DMA. ^d^ Data not available for film brittleness. ^e^ Data averaged in the range of 100–150 °C. ^f^ Data for the TA-26NA/*p*-PDA system. ^g^ Data for the TA-44BPHB/*p*-PDA(75);4,4′-ODA(25) copolymer system [21]. 
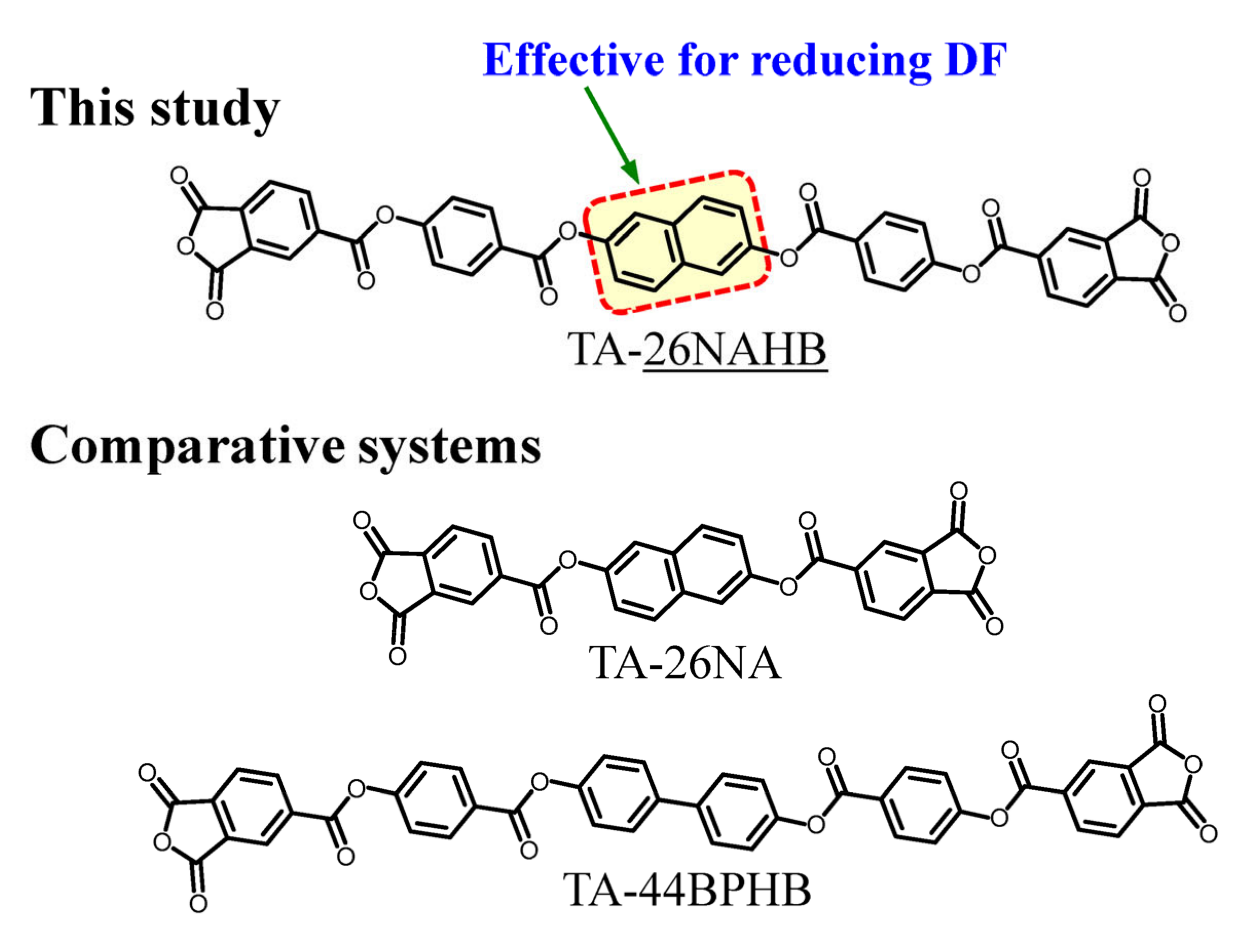

**Table 6:** Film properties of PEsIs derived from TA-HQHNA (*N*_Ar_ = 7) with common aromatic diamines. The structure of TA-HQHNA is shown at the bottom of this table.

No.	Diamine	*η*_inh_ PAA (dL g^−1^)	*T*_g_ (°C)	CTE (ppm K^−1^)	*E* (GPa)	*ε*_b_ av/max (%)	*σ*_b_ (GPa)	*T*_d_^5^ in N_2_ (°C)	*T*_d_^5^ in Air (°C)	*W*_A_ (%)	*ε*_r_ @10 GHz	tan *δ* @10 GHz
6	*p*-PDA	1.95	373 ^a^ 411 ^b^	1.9	6.50	3.7 6.4	0.14	458	446	0.21	2.97	0.00347
7	4,4′-ODA	1.34	331 ^a^ 335 ^b^ (178) ^c^	27.2 (23.3) ^d^	4.01	6.3 11.0	0.11	457	432	0.26	3.50	0.00189

^a^ *T*_g_ determined by DMA. ^b^ *T*_g_ determined by TMA. ^c^ *T*_β_ determined by DMA. ^d^ Data averaged in the range of 100–150 °C. 
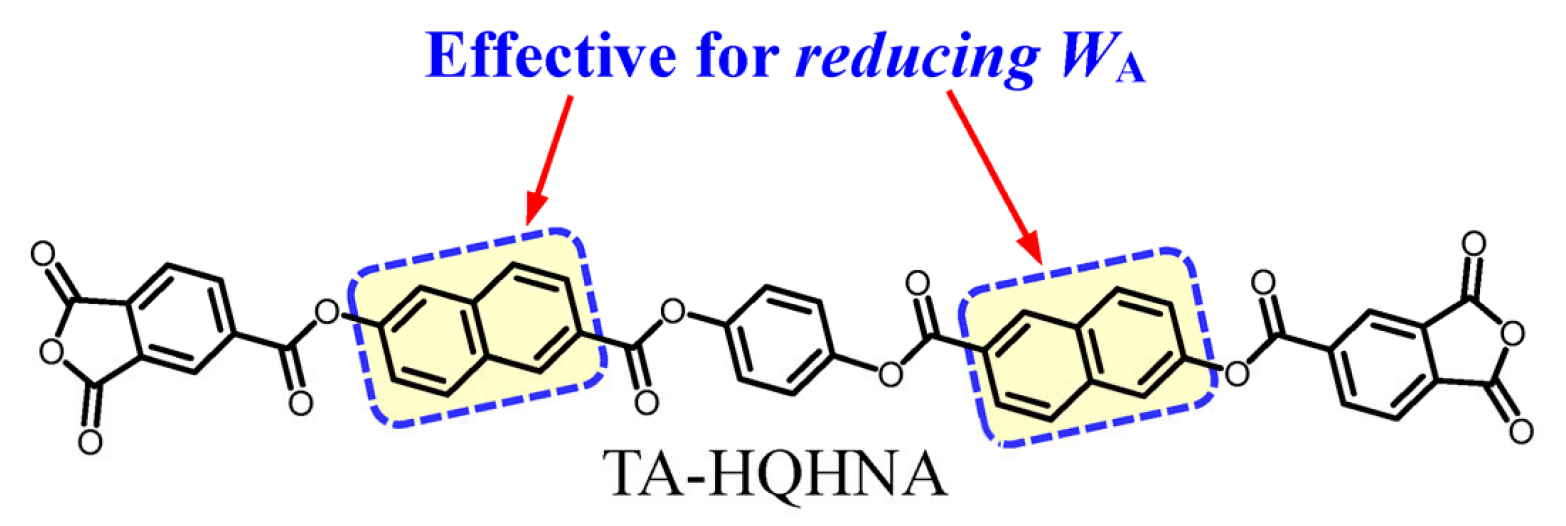

**Table 7:** Film properties of PEsIs derived from TA-26NAHNA (*N*_Ar_ = 8) with common aromatic diamines. The structure of TA-26NAHNA and the photographs of the PEsI films (#10, #11, and #12) are shown at the bottom of this table.

No.	Diamine (mol%)	*η*_inh_ PAA (dL g^−1^)	*T*_g_ (°C)	CTE (ppm K^−1^)	*E* (GPa)	*ε*_b_ av/max (%)	*σ*_b_ (GPa)	*T*_d_^5^ in N_2_ (°C)	*T*_d_^5^ in Air (°C)	*W*_A_ (%)	*ε*_r_ @10 GHz	tan *δ* @10 GHz
8	*p*-PDA	1.24	371 ^a^ 418 ^b^	3.8	7.70	2.4 3.7	0.14	467	461	0.26	3.20	0.00246
9	4,4′-ODA	0.86	342 ^a^ 309 ^b^ (169) ^c^	58.3 (18.7) ^d^	4.18	6.7 12.5	0.11	454	441	0.27	3.36	0.00170
10	APAB	0.83	358 ^a^ 366 ^b^ (165) ^c^	11.3 (8.5) ^d^	4.43	2.8 4.7	0.08	450	436	0.24	3.05	0.00128
11	M-APAB	1.07	365 ^a^ 373 ^b^ (178) ^c^	6.8 (6.3) ^d^	4.48	3.7 5.0	0.11	433	419	0.24	3.16	0.00128
12	M-APAB (70) BAPP (30)	0.83	349 ^a^ 373 ^b^ (182) ^c^	16.0 (13.9) ^d^	4.48	4.5 7.2	0.13	436	420	0.22	2.83	0.00138

^a^ *T*_g_ determined by DMA. ^b^ *T*_g_ determined by TMA. ^c^ *T*_β_ determined by DMA. ^d^ Data averaged in the range of 100–150 °C. 
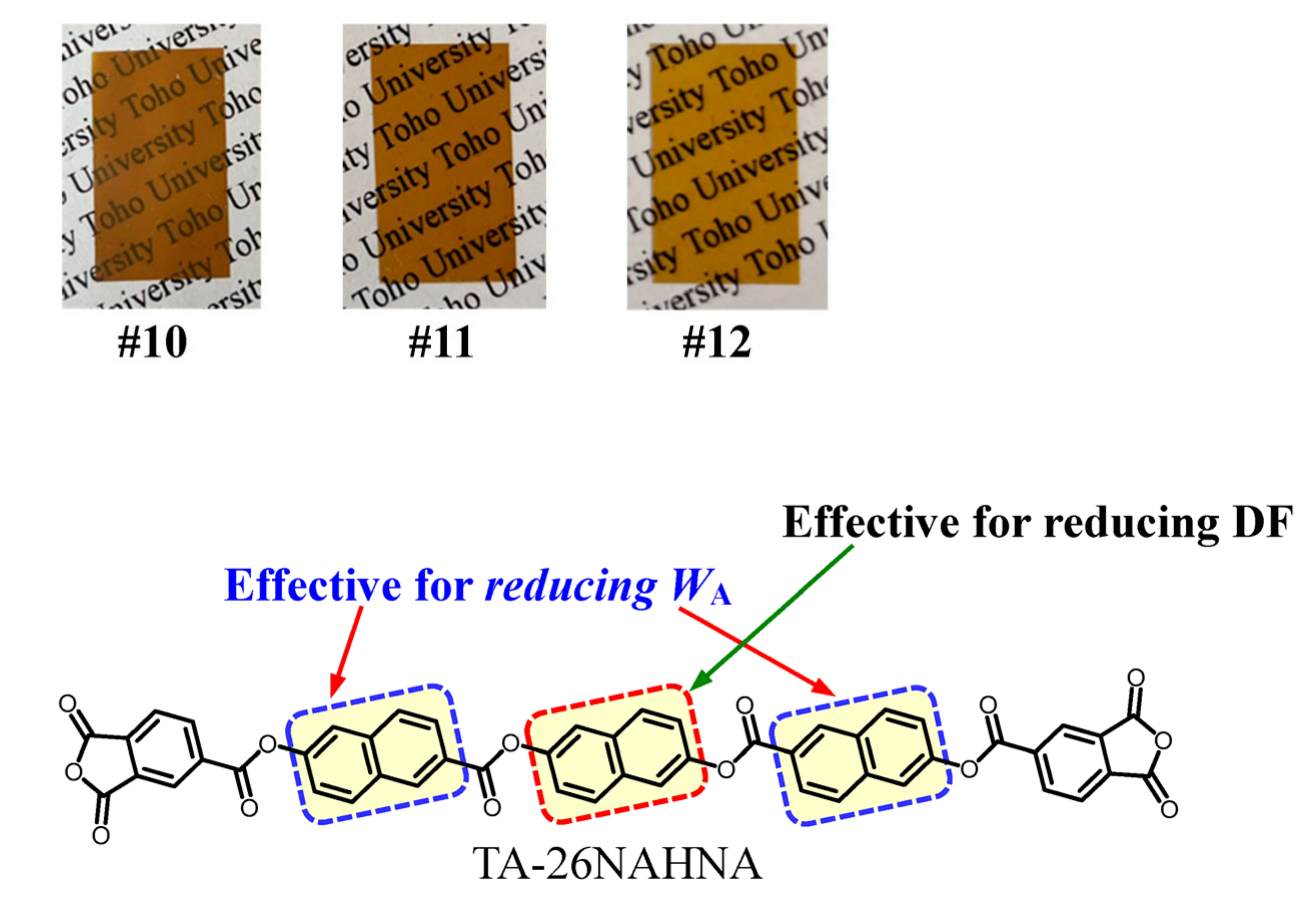

**Table 8:** Criteria for estimating the performance balance of the PEsIs for use in the dielectric substrates of high-performance FPCs.

Properties	Parameters	Relative Rank				
		1	2	3	4	5
Heat resistance	*T*_g_ (°C)	≤210	220–250	260–290	300–330	≥360 or ND ^a^
Low thermal expansion property	CTE (ppm K^−1^)	≥70	60–50	45–35	30–20	≤10
Low water uptake	*W*_A_ (%)	≥3.0	2.5–2.0	1.5–1.0	0.6–0.3	≤0.1
Toughness	*ε*_b max_ (%)	No film-forming ability or ≤2	5–10	20–30	40–60	≥80
Haze	haze (%)	≥50	40–30	25–20	15–10	≤5
Low DK in GHz range ^b^	*ε*_r_	≥3.7	3.6–3.4	3.3–3.1	3.0–2.8	≤2.6
Low DF in GHz range ^b^	tan *δ*	≥0.02	0.01–0.008	0.007–0.005	0.004–0.002	≤0.001

^a^ Non-detected *T*_g_ up to 450 °C on DMA. ^b^ Data measured at 40–60%RH and 20–30 °C.

## Data Availability

The data supporting this study are available within the article and/or in the Supplementary Materials.

## Supplementary Materials

The following supporting information can be downloaded at https://www.mdpi.com/article/10.3390/polym16050653/s1, Figure S1: DMA curves of the TA-26NAHB/*p*-PDA PEsI film. Figure S2: DMA (a) and TMA curves (b) of the TA-26NAHB/*p*-PDA(75);4,4′-ODA (25) PEsI film. Figure S3: Photographs of the PEsI films: (a) TA-TFMBHPBA/*p*-PDA (#1), (b) TA-26NAHB/*p*-PDA (#3), (c) TA-26NAHB/4,4′-ODA (#4), (d) TA-26NAHB/*p*-PDA(75);4,4′-ODA(25) (#5), (e) TA-HQHNA/*p*-PDA (#6), (f) TA-HQHNA/4,4′-ODA (#7).
