# Supplementary material for: Poly(ester imide)s with Low Linear Coefficients of Thermal Expansion and Low Water Uptake (VII): A Strategy to Achieve Ultra-Low Dissipation Factors at 10 GHz
Source: Polymers (Basel). 2024 Feb 28;16(5):653. doi: 10.3390/polym16050653 (PMC10934693; doi:10.3390/polym16050653)
Supplement: Supplementary file 1 [file polymers-16-00653-s001.zip › polymers-2849213-supplementary.pdf]

Supplementary Materials (Polymers-2849213, R1)

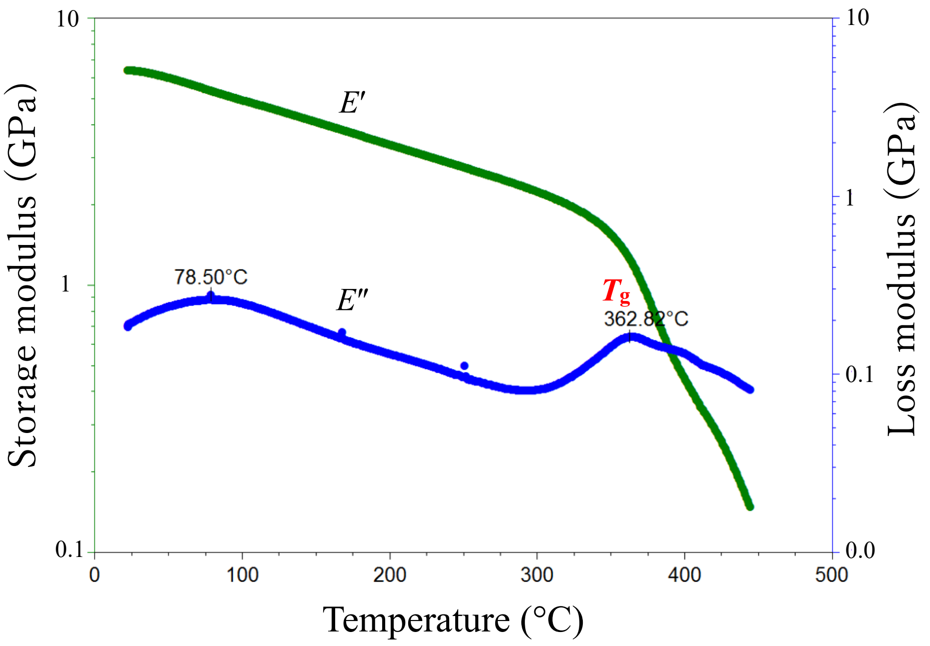

Figure S1. DMA curves of the TA-26NAHB/*p*-PDA PEsI film.

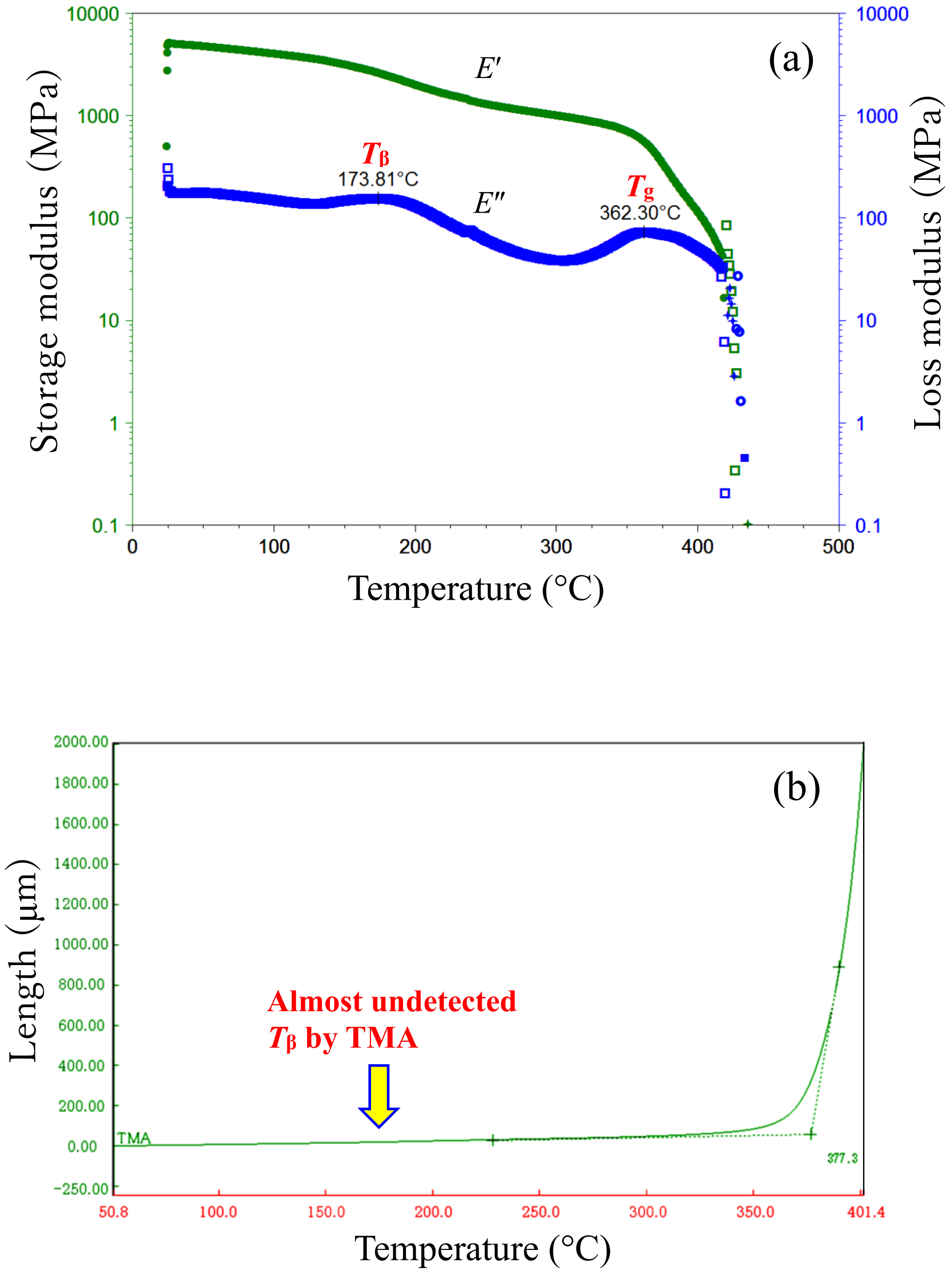

Figure S2. DMA (a) and TMA curves (b) of the TA-26NAHB/*p*-PDA(75);4,4′-ODA (25) PEsI film.

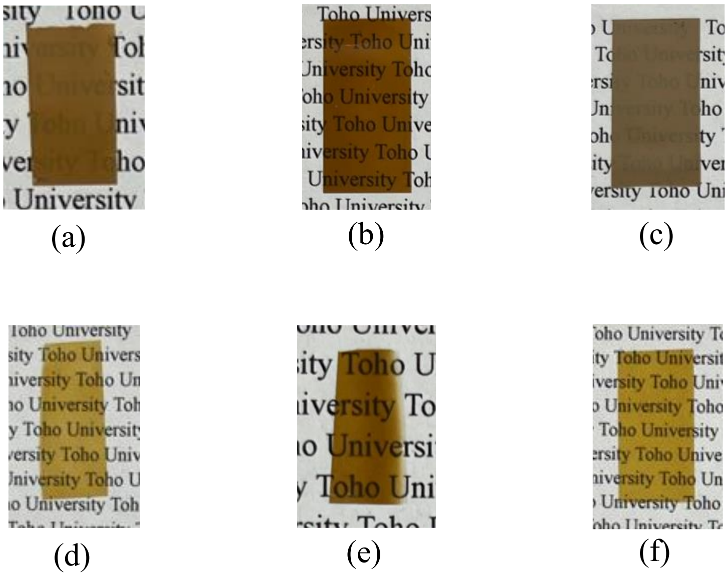

Figure S3. Photographs of the PEsI films: (a) TA-TFMBHPBA/*p*-PDA (#1), (b) TA-26NAHB/*p*-PDA (#3), (c) TA-26NAHB/4,4′-ODA (#4), (d) TA-26NAHB/*p*-PDA(75);4,4′-ODA(25) (#5), (e) TA-HQHNA/*p*-PDA (#6), (f) TA-HQHNA/4,4′-ODA (#7).
